# Supplementary material for: Cationic amphiphilic antihistamines inhibit STAT3 via Ca2+-dependent lysosomal H+ efflux
Source: Cell Rep. 2023 Feb 17;42(2):112137. doi: 10.1016/j.celrep.2023.112137 (PMC9989825; doi:10.1016/j.celrep.2023.112137)
Supplement: Document S1. Figures S1–S6 and Table S1 [file mmc1.pdf]

Cell Reports, Volume *42*

Supplemental information

Cationic amphiphilic antihistamines inhibit STAT3 via Ca^{2+}-dependent lysosomal H^+ efflux

Bin Liu, Ran Chen, Yidan Zhang, Jinrong Huang, Yonglun Luo, Susanne Rosthøj, Chenyang Zhao, and Marja Jäättelä

Supplementary Information

Figure S1. CADs induce rapid cytosolic acidification in cancer cells. Related to figure 1.

A. Loss of plasma membrane integrity of HeLa cells treated with 6 µM terfenadine or 15 µM ebastine for indicated times, stained with membrane-permeable Hoechst (all cells) and propidium iodide (cells with plasma membrane permeabilization) and analyzed by Celigo Imaging Cytometer.

B. Kinetics of SypHer3s FLIs in HeLa cells left untreated or treated with 6 µM terfenadine or 15 µM ebastine for indicated times and analyzed as in Figure 1A.

C. Representative images of Ebastine-treated HeLa cells presented in Figure 1A (*right* panel) with arrows pointing to cells with typical CAD-induced changes. Scale bars, 30 µM.

D. SypHer3s FLI in HeLa-SypHer3s cells left untreated or treated with 10 µM niclosamide (positive control for cytosolic acidification), 6 µM terfenadine, 15 µM ebastine, 10 µM astemizole or 6 µM penfluridol for 1 h prior to the flow cytometry analysis.

E. SypHer3s FLI in A549-SypHer3s cells treated for 2 h with DMSO, 10 µM niclosamide (positive control), 6 µM terfenadine, or 15 µM ebastine and analyzed by flow cytometry.

F. Quantification of LGALS1/galectin 1 puncta (leaky lysosomes) in A549 cells treated with 6 µM terfenadine or 15 µM ebastine for 0 - 8 h.

Error bars, SD of three triplicate independent experiments with ≥ 10000 (A, D, and E) or ≥10 (B and F) randomly chosen cells analyzed in each sample. *, $P < 0.05$; **, $P < 0.01$; ***, $P < 0.001$ as analyzed by one-way Anova with Tukey multiple comparison (D and E) or two-way Anova with Dunnet multiple comparison (A, B and F).

Figure S2. CAD-induced cytosolic acidification depends on P2RX4 but not cAMP generation. Related to figure 3.

A, B. Cytosolic pH values of MCF7-SypHer3s cells treated with indicated siRNAs for 72 and with DMSO or 6 µM terfenadine for the last 2 h (*left*) were analyzed as in Figure 1C. Representative (n=3) immunoblots of indicated proteins in siRNA-treated MCF7-SypHer3S cells (*right*).

C. Flamindo (*left*) and SypHer3s (*right*) FLIs in HeLa cells treated with indicated concentrations of drugs for 1 h (*left*) or 2 h (*right*) and analyzed by flow cytometry.

Error bars, SD of three triplicate independent experiments with ≥10 (A, B) or ≥ 10000 (C) randomly chosen cells analyzed in each sample. *, $P < 0.05$; **, $P < 0.01$; ***, $P < 0.001$ as analyzed by one-way Anova with Tukey multiple comparison (C) or two-way Anova with Dunnet multiple comparison (A, B).

Figure S3. CADs trigger lysosomal translocation of STAT3. Related to figure 4.

Representative images of A549 triple cells with RFP-tagged endogenous STAT3 transfected with LAMP1-BFP and treated with DMSO, 6 µM terfenadine, or 15 µM ebastine for 2 h. Scale bars, 10 µm. For quantification see Figure 4B.

Figure S4. CADs reduce Y705-STAT3 phosphorylation and CCND1 expression. Related to figure 5.

A. Quantification of P-S727-STAT3 / STAT3 and STAT3 / TUBA1 ratios in immunoblots of lysates of HeLa cells left untreated or treated with 6 µM terfenadine, 15 µM ebastine, 10 µM astemizole or 6 µM penfluridol for indicated times. See Figure 5A for representative blots.

B-E. Representative immunoblots of indicated proteins from A549 (B) and PANC1 (D) cells treated with CADs as indicated and the quantification of blots from three independent experiments (C and E).

F. Quantification of the amount of dephosphorylated STAT3 according to Fig 5F.

Error bars, SD of three independent experiments. *, $P < 0.05$; **, $P < 0.01$; ***, $P < 0.001$ as analyzed by by two-way Anova with Dunnet multiple comparison.

Figure S5. Examples of terfenadine- and ebastine-regulated STAT3 target genes. Related to figure 6.

A and B. Box plots for the expression of indicated STAT3 target genes in HeLa cells treated with 6 µM terfenadine (A) or 15 µM ebastine (B) for 0, 2, or 8 h. Boxes, median and interquartile range of the values from three independent RNASeq analyses. Whiskers, variability outside the quartiles. TPM, transcripts per million.

C and D. KEGG enrichment analysis of terfenadine and ebastine specifically-downregulated STAT3 target genes. Fisher's test p values are displayed.

Significance of statistics is indicated when fold change is bigger than 1.5 and adjusted P value meet criteria: *, $P < 0.05$; **, $P < 0.01$; ***, $P<0.001$. Data is based on three independent RNAseq experiments.

Figure S6. CAD-induced cytosolic acidification exerts a synergistic anti-tumor effect with WP1066. Related to figure 7.

A. Representative immunoblots of indicated proteins in HeLa cells with indicated treatments (*left*) and the quantification of blots from three independent experiments (*right*).

B. Luciferase activity in the media of A549 triple cells expressing CCND1 promoter-driven secreted luciferase. Cells were left untreated or treated with 4 µM terfenadine, or 8 µM ebastine for indicated times.

C. Cell death of HeLa cells with indicated treatments for 24 h determined by propidium iodide and Hoechst-33342 staining employing Celígo Imaging Cytometer.

D. Cell death of A549 cells with indicated treatments for 24 h determined by propidium iodide and Hoechst-33342 staining employing Celígo Imaging Cytometer. Synergy score was determined by the Bliss independent model (See methods).
E. SypHer3s FLI in HeLa cells treated by 4 µM terfenadine, 5 µM WP1066, or the combination of both drugs for 1 h and analyzed by FACS.
Error bars, SD of three independent triplicate experiments with ≥1000 cells analyzed in each condition.
*, $P < 0.05$; **, $P < 0.01$; ***, $P < 0.001$ as analyzed by one way Anova with Turkey multiple comparison (E) or two-way Anova with Dunnet multiple comparison (A-D).

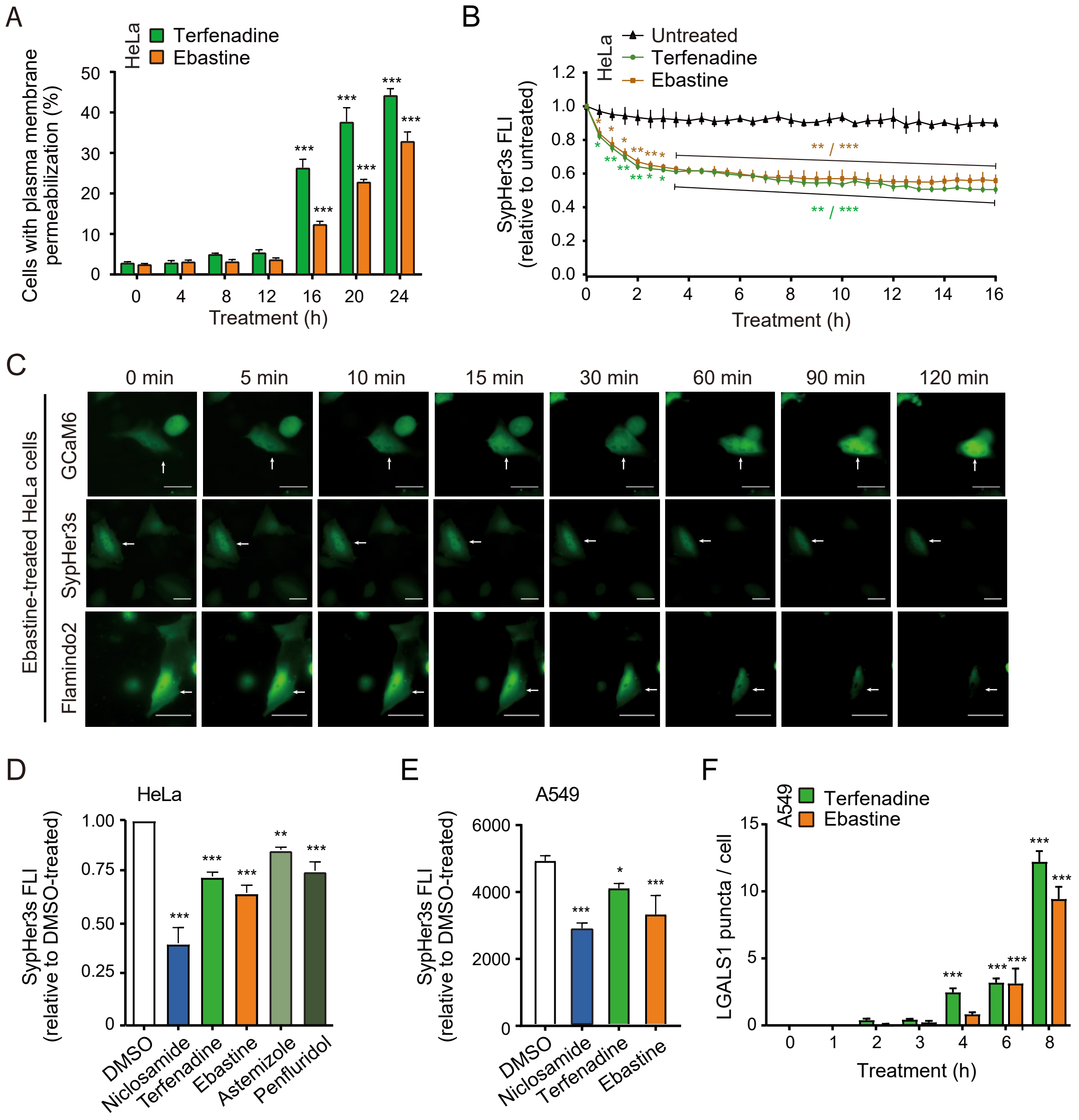
A
HeLa
Terfenadine
Ebastine
Cells with plasma membrane permeabilization (%)
Treatment (h)
B
HeLa
Untreated
Terfenadine
Ebastine
SypHer3s FLI (relative to untreated)
Treatment (h)
C
0 min
5 min
10 min
15 min
30 min
60 min
90 min
120 min
Ebastine-treated HeLa cells
GCaM6
SypHer3s
Flamindo2
D
HeLa
SypHer3s FLI (relative to DMSO-treated)
DMSO
Niclosamide
Terfenadine
Ebastine
Astemizole
Penfluridol
E
A549
SypHer3s FLI (relative to DMSO-treated)
DMSO
Niclosamide
Terfenadine
Ebastine
F
A549
Terfenadine
Ebastine
LGALS1 puncta / cell
Treatment (h)

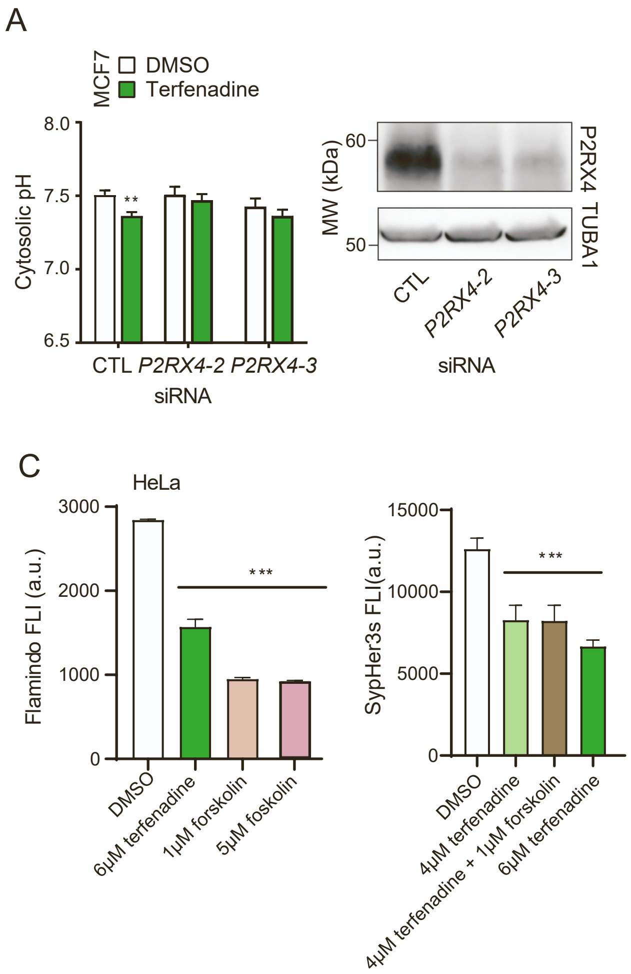
A
MCF7
DMSO
Terfenadine
Cytosolic pH
8.0
7.5
7.0
6.5
**
CTL P2RX4-2 P2RX4-3
siRNA
MW (kDa)
60
50
P2RX4
TUBA1
CTL
P2RX4-2
P2RX4-3
siRNA
B
MCF7
DMSO
Tefenadine
Cytosolic pH
8.0
7.5
7.0
6.5
**
**
**
CTL P2RX7-2 P2RX7-3
MW (kDa)
60
40
P2RX7
ATCB
CTL
P2RX7-2
P2RX7-3
siRNA
C
HeLa
Flamindo FLI (a.u.)
3000
2000
1000
0
***
DMSO
6μM terfenadine
1μM forskolin
5μM foskolin
SypHer3s FLI(a.u.)
15000
10000
5000
0
***
DMSO
4μM terfenadine
4μM terfenadine + 1μM forskolin
6μM terfenadine

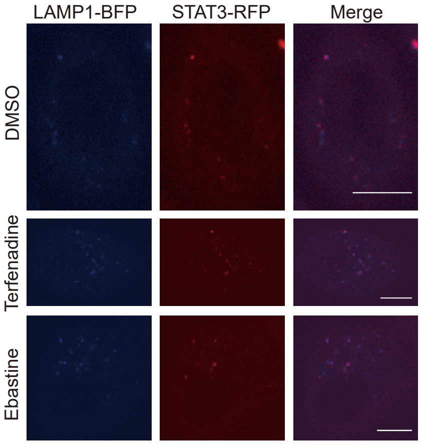
LAMP1-BFP
STAT3-RFP
Merge
DMSO
Terfenadine
Ebastine

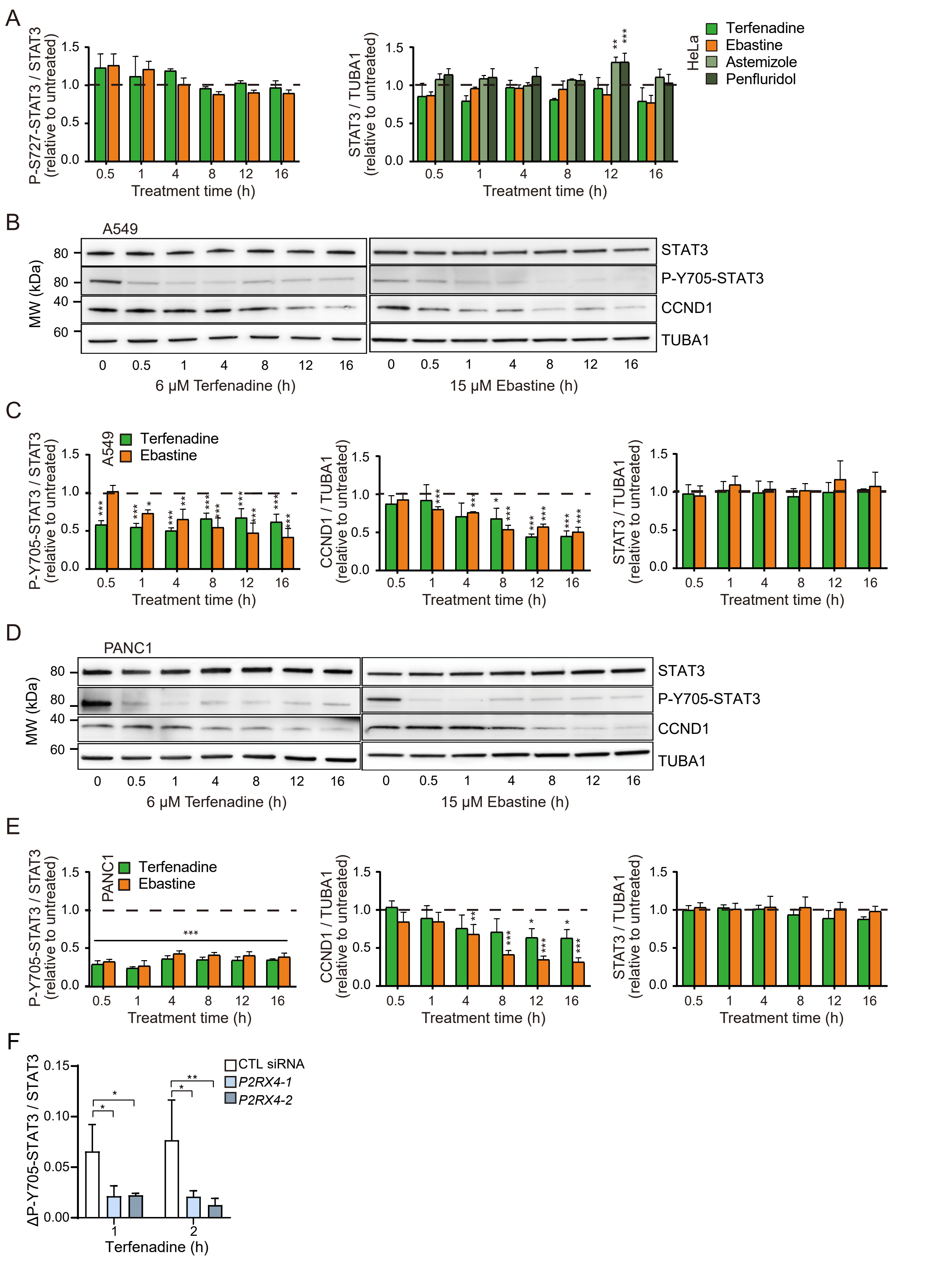
A
P-S727-STAT3 / STAT3 (relative to untreated)
Treatment time (h)
STAT3 / TUBA1 (relative to untreated)
HeLa
Terfenadine
Ebastine
Astemizole
Penfluridol
B
A549
MW (kDa)
STAT3
P-Y705-STAT3
CCND1
TUBA1
6 µM Terfenadine (h)
15 µM Ebastine (h)
C
P-Y705-STAT3 / STAT3 (relative to untreated)
CCND1 / TUBA1 (relative to untreated)
D
PANC1
E
F
ΔP-Y705-STAT3 / STAT3
CTL siRNA
P2RX4-1
P2RX4-2
Terfenadine (h)

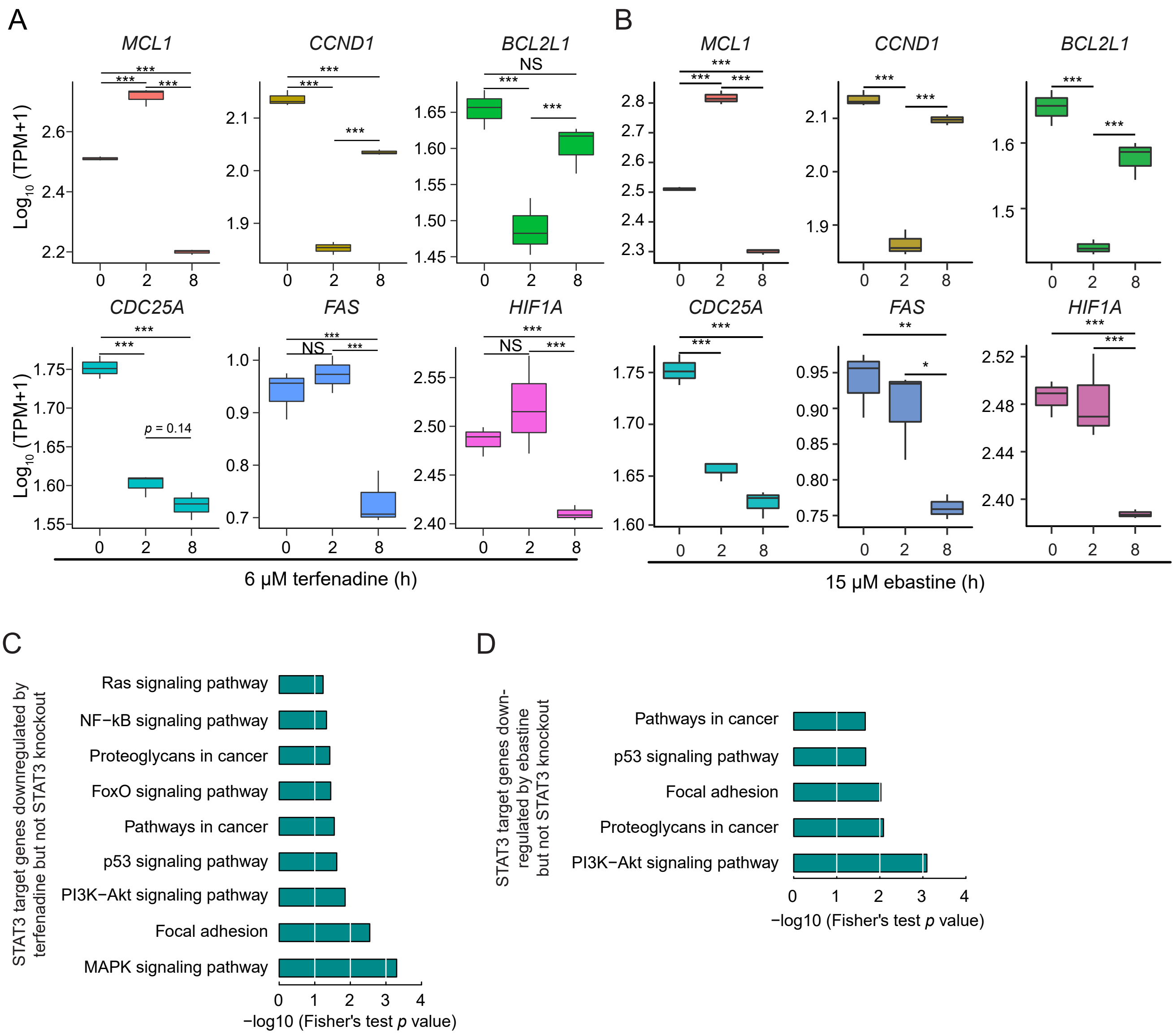
A
B
MCL1
CCND1
BCL2L1
CDC25A
FAS
HIF1A
Log10 (TPM+1)
NS
p = 0.14
6 μM terfenadine (h)
15 μM ebastine (h)
C
D
STAT3 target genes downregulated by terfenadine but not STAT3 knockout
Ras signaling pathway
NF−kB signaling pathway
Proteoglycans in cancer
FoxO signaling pathway
Pathways in cancer
p53 signaling pathway
PI3K−Akt signaling pathway
Focal adhesion
MAPK signaling pathway
−log10 (Fisher's test p value)
STAT3 target genes down-regulated by ebastine but not STAT3 knockout
Pathways in cancer
p53 signaling pathway
Focal adhesion
Proteoglycans in cancer
PI3K−Akt signaling pathway
−log10 (Fisher's test p value)

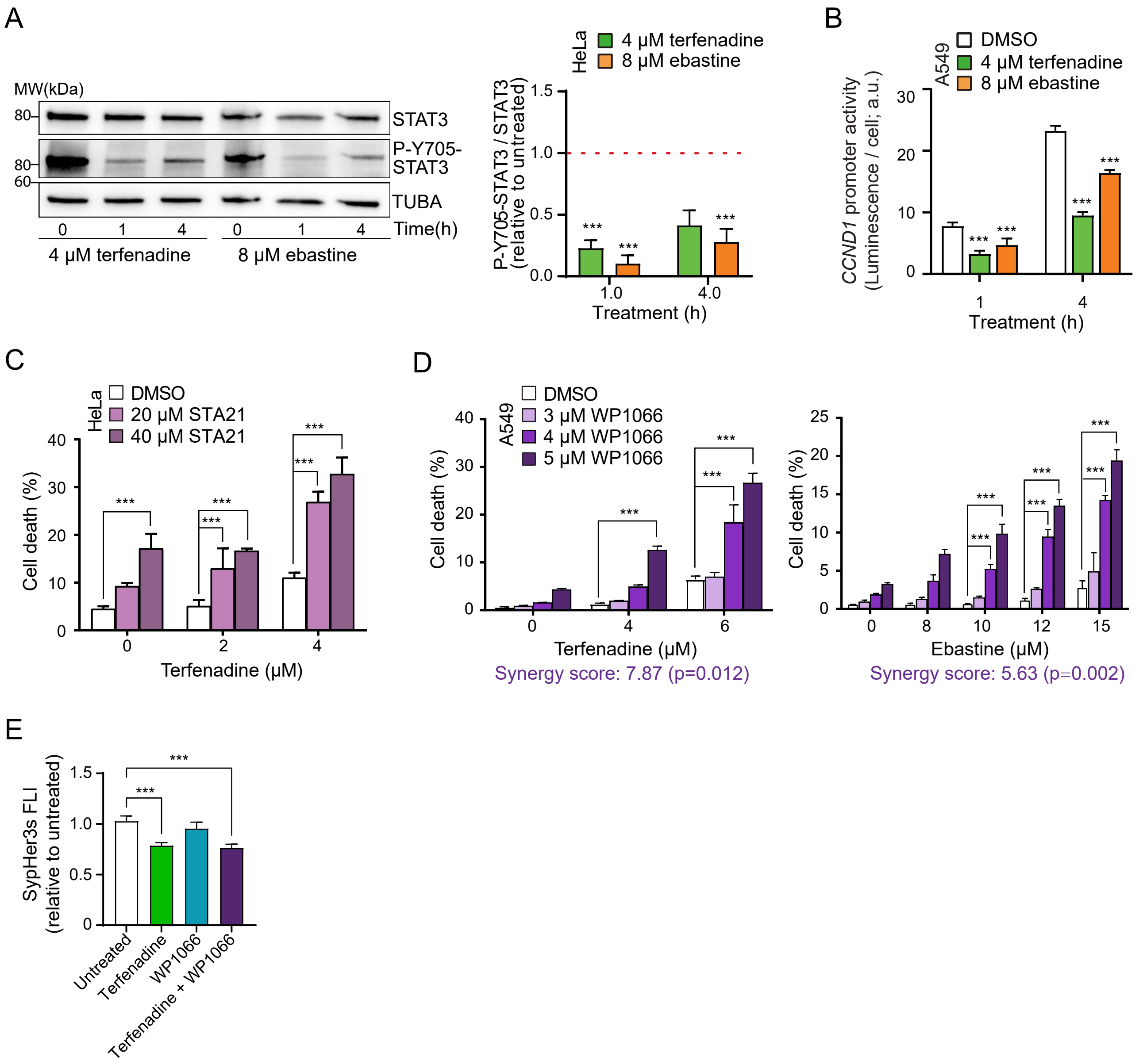
A
MW(kDa)
80
80
60
STAT3
P-Y705-STAT3
TUBA
0 1 4 0 1 4 Time(h)
4 μM terfenadine
8 μM ebastine
HeLa
4 μM terfenadine
8 μM ebastine
P-Y705-STAT3 / STAT3 (relative to untreated)
Treatment (h)
B
A549
DMSO
4 μM terfenadine
8 μM ebastine
CCND1 promoter activity (Luminescence / cell; a.u.)
Treatment (h)
C
HeLa
DMSO
20 μM STA21
40 μM STA21
Cell death (%)
Terfenadine (μM)
D
A549
DMSO
3 μM WP1066
4 μM WP1066
5 μM WP1066
Cell death (%)
Terfenadine (μM)
Synergy score: 7.87 (p=0.012)
Cell death (%)
Ebastine (μM)
Synergy score: 5.63 (p=0.002)
E
SypHer3s FLI (relative to untreated)
Untreated
Terfenadine
WP1066
Terfenadine + WP1066

Table S1 Oligos and siRNAs

qPCR primers	CCND1S	GAAGATCGTCGCCACCTG
	CCND1AS	GACCTCCTCCTCGCACTTCT
	ACTINS	TGACTTAGTTGCGTTACACCCTT
	ACTINAS	CACCTTCACCGTTCCAGTTTT
Cloning primers	mCherryCASBamHI	gc GGATCC tca CTTGTACAGCTCGTCCATGC
	mCherryNSXhoI	gc CTCGAG ATGGTGAGCAAGGGCGAGGAG
	TMEM165NSKozakNheI	gc GCTAGC gccacc ATGGCGGCCGCGGCTCCAGG
	TMEM165CASXhoI	gc CTCGAG AAAACCAGAATCAGGGCTTAT
	mKeimaNSBamHI	gc GGATCC atggtgagcgtgatcgccaag
	mKeimaCASXbaI	GC TCTAGA TTAGCCCAGCAGGGAGTGGCGG
siRNA	STAT3-1	GAAUCACGCCUUCUACAGA/UCUGUAGAAGGCGUGAUUC
	P2RX4-2	CAAGUCGUGCAUUUAUGAUtt/AUCAUAAAUGCACGACUUGtt
	P2RX4-3	GUCCUCUACUGCAUGAAGAtt/UCUUCAUGCAGUAGAGGACtt
	TMEM165-1	GUAUCUGAAUUGGGUGAUAtt/UAUCACCCAAUUCAGAUACaa
	TMEM165-5	CAGGGUCUAUACAUACUAUtt/AUAGUAUGUAUAGACCCUGaa
	TMEM165-8	GCAUAACAGUACCUCAGAAtt/UUCUGAGGUACUGUUAUGCtt
