# Supplementary material for: Cationic amphiphilic antihistamines inhibit STAT3 via Ca2+-dependent lysosomal H+ efflux
Source: Cell Rep. 2023 Feb 17;42(2):112137. doi: 10.1016/j.celrep.2023.112137 (PMC9989825; doi:10.1016/j.celrep.2023.112137)
Supplement: Document S2. Article plus supplemental information [file mmc6.pdf]

Cell Reports

Article

Cationic amphiphilic antihistamines inhibit STAT3 via Ca^{2+}-dependent lysosomal H^+ efflux

Graphical abstract

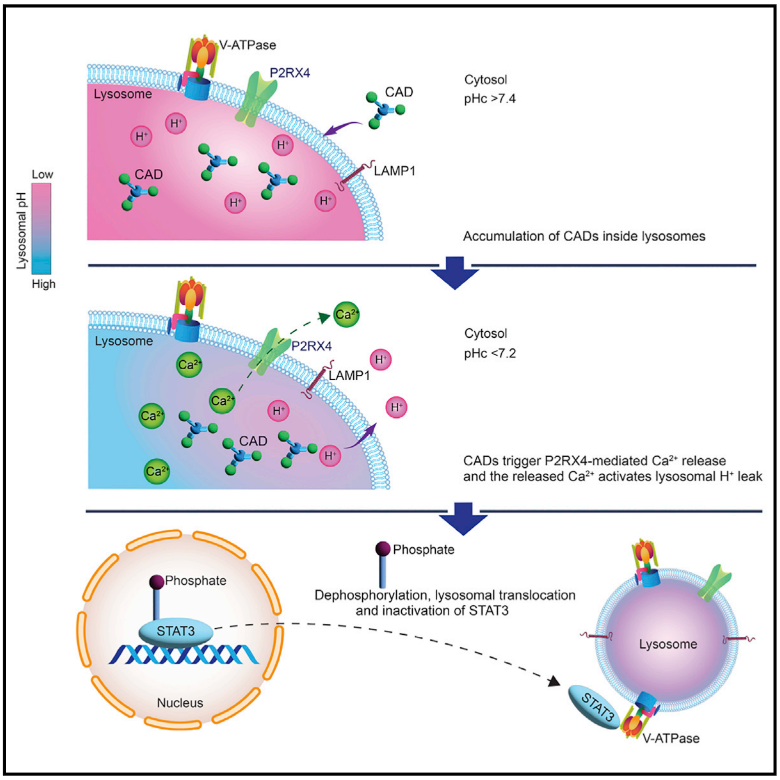

Highlights

- CADs trigger lysosomal H^+ efflux and cytosolic acidification
- CADs are effective STAT3 inhibitors
- CADs sensitize cancer cells to apoptosis induced by STAT3 inhibition
- CADs and STAT3 inhibitor WP1066 show synergistic anticancer activity *in vivo*

Authors

Bin Liu, Ran Chen, Yidan Zhang, ..., Susanne Rosthøj, Chenyang Zhao, Marja Jäättelä

Correspondence

liu@cancer.dk (B.L.),
mj@cancer.dk (M.J.)

In brief

Liu et al. find that cationic amphiphilic drugs induce a rapid lysosome H^+ efflux that is dependent on P2RX4-mediated lysosomal Ca^{2+} release and precedes lysosome membrane permeabilization. This mechanism can be applied together with STAT3 inhibition to exert synergistic antitumor activity both *in vitro* and *in vivo*.

Liu et al., 2023, Cell Reports *42*, 112137
February 28, 2023 © 2023 The Authors.
https://doi.org/10.1016/j.celrep.2023.112137

CellPress

Cell Reports

CellPress
OPEN ACCESS

Article

Cationic amphiphilic antihistamines inhibit STAT3 via Ca^{2+}-dependent lysosomal H^+ efflux

Bin Liu,[1,*] Ran Chen,[1] Yidan Zhang,[2] Jinrong Huang,[3,4,5] Yonglun Luo,[3,5,6] Susanne Rosthøj,[7] Chenyang Zhao,[2] and Marja Jäättelä[1,8,9,*]

[1]Cell Death and Metabolism, Center for Autophagy, Recycling and Disease (CARD), Danish Cancer Society Research Center (DCRC), 2100 Copenhagen, Denmark
[2]School of Medicine and Pharmacy, Ocean University of China, Qingdao 266555, China
[3]BGI-Shenzhen, Shenzhen 518083, China
[4]Department of Biology, University of Copenhagen, 2200 Copenhagen, Denmark
[5]Lars Bolund Institute of Regenerative Medicine, Qingdao-Europe Advanced Institute for Life Sciences, BGI-Qingdao, Qingdao 266555, China
[6]Department of Biomedicine, Aarhus University, 8000 Aarhus, Denmark
[7]Statistics and Data Analysis, Danish Cancer Society Research Center, Copenhagen, Denmark
[8]Department of Cellular and Molecular Medicine, Faculty of Health Sciences, University of Copenhagen, 2200 Copenhagen, Denmark
[9]Lead contact
*Correspondence: liu@cancer.dk (B.L.), mj@cancer.dk (M.J.)
https://doi.org/10.1016/j.celrep.2023.112137

SUMMARY

Commonly used antihistamines and other cationic amphiphilic drugs (CADs) are emerging as putative cancer drugs. Their unique chemical structure enables CADs to accumulate rapidly inside lysosomes, where they increase lysosomal pH, alter lysosomal lipid metabolism, and eventually cause lysosomal membrane permeabilization. Here, we show that CAD-induced rapid elevation in lysosomal pH is caused by a lysosomal H^+ efflux that requires P2RX4-mediated lysosomal Ca^{2+} release and precedes the lysosomal membrane permeabilization. The subsequent cytosolic acidification triggers the dephosphorylation, lysosomal translocation, and inactivation of the oncogenic signal transducer and activator of transcription 3 (STAT3) transcription factor. Moreover, CAD-induced lysosomal H^+ efflux sensitizes cancer cells to apoptosis induced by STAT3 inhibition and acts synergistically with STAT3 inhibition in restricting the tumor growth of A549 non-small cell lung carcinoma xenografts. These findings identify lysosomal H^+ efflux and STAT3 inhibition as anticancer mechanisms of CADs and reinforce the repurposing of safe and inexpensive CADs as cancer drugs with a drug combination strategy.

INTRODUCTION

Cancer cells are continuously exposed to various stresses due to the persistent activity of oncogenic pathways and hazardous cancer environments. Consequently, they become addicted to survival-promoting stress response pathways and to elevated activity of homeostasis-preserving cellular processes, whose targeting is opening new possibilities for cancer therapy.[1,2] Among the promising non-oncogene cancer targets is the endo-lysosomal compartment (hereafter referred to as lysosomes), which not only provides cancer cells with energy and essential macromolecules but also maintains several cancer characteristics such as growth signaling, angiogenesis, invasion, pH gradient reversal, and drug resistance.[3–6]

Lysosomes are membrane-surrounded acidic organelles containing over 50 soluble hydrolytic enzymes that serve as executors of cellular catabolism.[7] Lysosomal ion channels and transporters establish concentration gradients for H^+, Ca^{2+}, and other ions across the lysosomal membrane.[8,9] High H^+ concentration is a key physiological feature of lysosomes as it ensures the optimal activity of lysosomal hydrolyses and controls lysosomal membrane trafficking. The steep pH gradient between the slightly basic cytosol (pH 7–7.5) and acidic lysosomal lumen (pH 4.5–5) is maintained by vacuolar-type H^+-ATPase (V-ATPase), which is composed of a peripheral, ATP-hydrolyzing V_1 domain and a membrane-integral V_0 proton channel.[10] Besides acidifying the lysosomal lumen, V-ATPase assists plasma membrane-localized, acid-exporting ion pumps and exchangers in the removal of H^+ from the cytosol.[6,11] This is of special importance in cancer cells that generate large quantities of acid while depending on alkaline cytosol (pH ∼7.5) for tumor progression.[11] To ensure the maintenance of alkaline cytosol, cancer cells enhance the H^+ scavenging capacity of lysosomes upon cytosolic acidification. This is brought about by the recruitment of an oncogenic transcription factor, signal transducer and activator of transcription 3 (STAT3), to the lysosomal membrane, where it associates with V-ATPase and stimulates its ATPase activity.[12] Furthermore, cancer cells obtain a robust membrane trafficking system that contains numerous acidic organelles including *trans*-Golgi network and endosomes en route to

This is an open access article under the CC BY license (http://creativecommons.org/licenses/by/4.0/).

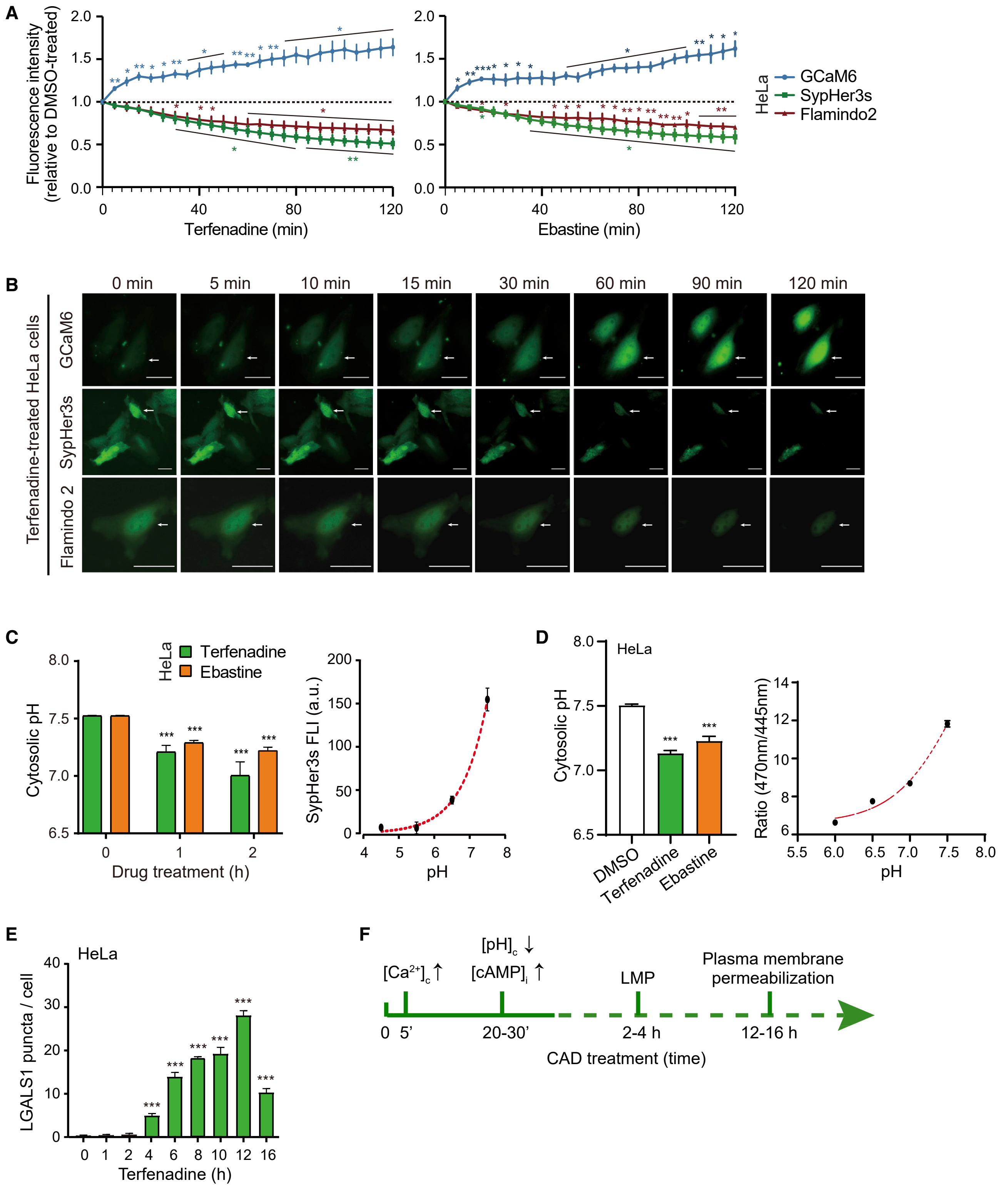

Figure 1. CADs induce rapid cytosolic acidification in HeLa cells

(A) Kinetics of fluorescence intensities (FLIs) of SypHer3s (cytosolic pH indicator), GCaM6 ($[Ca^{2+}]c$ indicator), and Flamindo2 (inverted $[cAMP]_i$ indicator) in HeLa cells treated with 6 μM terfenadine or 15 μM ebastine as indicated. Images were taken by ImageXpress Micro Confocal imaging system every 5 min and analyzed by MetaXpress image acquisition and analysis software. See Figure S1B for 16 h kinetics of SypHer3S fluorescence in untreated and CAD-treated HeLa cells.

(legend continued on next page)

lysosomes, which can release their acidic contents via exocytosis. As a result, H^+ constantly scavenged during lysosome maturation and reformation flows dynamically and continuously to extracellular space.[6] Such a mechanism substantially expands lysosomal capacity to control pH homeostasis. Notably, lysosomal patch-clamp experiments have revealed that the H^+ flux across the lysosomal membrane is bidirectional.[8,13–15] In living cells, the basal lysosomal H^+ leak has, however, been demonstrated only indirectly by showing an increase in lysosomal pH after the inhibition of the lysosomal V-ATPase activity,[16] while genetic and pharmacological ways to modulate lysosomal H^+ efflux remain to be identified.

Increasing the luminal pH is an efficient way to disturb lysosomal function. This can be effectively achieved by cationic amphiphilic drugs (CADs), many of which are presently used to treat a wide range of diseases including allergies, psychiatric disorders, heart diseases, and infections.[4,17] Due to their chemical properties, CADs diffuse freely through cellular membranes and accumulate in acidic lysosomes, where they can reach up to 1,000-fold concentrations due to the protonation of their amine groups and subsequent ion trapping.[18] The effective protonation of CADs increases lysosomal pH and activates a pH-sensitive P2X purinergic receptor 4 (P2RX4) to release lysosomal Ca^{2+}, which then activates the synthesis of cAMP in the cytosol.[19] Together with CAD-induced changes in lysosomal lipid catabolism, cytosolic cAMP contributes to the CAD-induced lysosomal membrane permeabilization, which in addition to completely dissipating lysosomal pH gradient also allows lysosomal hydrolases to leak to the cytosol, where they serve as executors of lysosome-dependent cell death.[19–21] Based on the ability of CADs to inhibit cancer-promoting lysosomal functions, induce lysosome-dependent cell death specifically in cancer cells, sensitize cancer cells to chemotherapy, and inhibit cancer growth in numerous animal models,[4,20,22–27] the interest in repurposing CADs for cancer therapy is rapidly increasing. This interest is further encouraged by the emerging pharmacoepidemiologic data showing a strong correlation between the post-diagnostic use of CADs, especially cationic amphiphilic antihistamines, and reduced cancer mortality among patients with cancer in Denmark and Sweden.[28–31]

Prompted by the ability of clinically relevant CAD antihistamines to induce cytosolic acidification in cancer cells, we carefully compared the kinetics of the CAD-induced cytosolic acidification with that of CAD-induced lysosomal Ca^{2+} release, increase in cytosolic cAMP, and lysosomal membrane permeabilization. Surprisingly, we found that CAD-induced cytosolic acidification occurred already shortly after the lysosomal Ca^{2+} release and 1.5–3.5 h before any signs of lysosomal membrane permeabilization were detectable. Further mechanistic studies demonstrated that the CAD-induced and P2RX4-mediated lysosomal Ca^{2+} release triggers two independent signaling pathways, one leading to lysosomal H^+ efflux and cytosolic acidification and the other one resulting in cAMP generation, lysosomal membrane permeabilization, and lysosome-dependent cell death. Given that cytosolic acidification is a potent inhibitor of the transcriptional activity of STAT3,[12] we then investigated the effect of CADs on the subcellular localization and phosphorylation status of STAT3 and the expression of STAT3 target genes and identified several clinically relevant CADs as potent inhibitors of STAT3. Finally, we demonstrated that the cytosolic acidification induced by sublethal concentrations of CADs significantly sensitized cancer cells to apoptosis induced by pharmacological or genetic inhibition of STAT3 and acted synergistically with STAT3 inhibition in restricting the growth of A549 non-small cell lung carcinoma xenografts in mice. Taken together, our data identify cytosolic acidification and STAT3 inhibition as novel anticancer mechanisms of clinically relevant CADs and provide a novel drug combination strategy for cancer therapy.

RESULTS

CADs induce rapid cytosolic acidification in cancer cells

As membrane-permeable weak bases, CADs accumulate rapidly in acidic lysosomes and increase their pH.[4] To investigate the consequences of lysosomal CAD accumulation on cytosolic pH (pH_c), we transfected HeLa cervix carcinoma cells with SypHer3s, a genetically encoded fluorescent pH probe capable of detecting pH_c changes in living cells.[32] We then treated the obtained HeLa-SypHer3s cells with two cationic amphiphilic antihistamines, terfenadine and ebastine, at concentrations that kill approximately 40% of the cells (LC_{40}) in 24 h (Figure S1A) and followed the SypHer3s fluorescence with ImageXpress Micro Confocal Imaging System for 2 h with 5 min intervals or for 16 h with 30 min intervals. These concentrations were chosen to obtain most reproducible data from the image-based cell death quantifications and to allow further studies with treatments that either sensitize or rescue the cells to CADs. Both drugs induced rapid acidification of the cytosol as indicated by significant decreases in SypHer3s fluorescence intensity already 30 min after the treatments (Figures 1A, 1B, S1B, and S1C). The fluorescence kept decreasing for an additional 2 h to reach steady-state levels approximately 40% below that in untreated cells (Figures 1A, 1B, S1B, and S1C). The SypHer3s fluorescence intensity in untreated cells corresponded to the pH_c of 7.5, while the estimated pH_c in cells treated for 2 h with terfenadine and ebastine were 7 and 7.2, respectively (Figure 1C). The

(B) Representative images of terfenadine-treated cells in (A) with arrows pointing to cells with typical CAD-induced changes. Scale bars, 30 μM. See Figure S1C for corresponding images of ebastine-treated cells.

(C) Cytosolic pH values of HeLa-SypHer3s cells treated as indicated (left) and analyzed by ImageXpress. A standard curve for SypHer3s FLI at pH ranging from 4.5 to 7.5 (right).

(D) Cytosolic pH values determined with ratiometric dye BCECF-AM and analyzed by ImageXpress. HeLa cells were treated with indicated drugs for 2 h.

(E) Quantification of LGALS1/galectin 1 puncta (leaky lysosomes) in HeLa cells treated with 6 μM terfenadine for 0–16 h.

(F) Timeline of the initiation of investigated CAD-induced events in HeLa cells.

Error bars, SD of three independent triplicate experiments with ≥10 randomly chosen cells analyzed in each sample. *p < 0.05; **p < 0.01; ***p < 0.001 as analyzed by one-way ANOVA with Tukey (A, D, and E) or two-way ANOVA with Dunnett (C) multiple comparison.

ability of CADs to acidify the cytosol was confirmed by using a ratiometric pH-sensitive dye BCECF-AM (Figure 1D). The treatment of HeLa cells with other clinically relevant CADs that are potent inducers of lysosome-dependent cancer cell death, i.e., astemizole (antihistamine) and penfluridol (antipsychotic),[28] induced similar cytosolic acidification when applied at concentrations around their LC_{40} (Figure S1D).

We have previously demonstrated that CADs trigger an early increase in the free cytosolic Ca^{2+} $[Ca^{2+}]_c$ and intracellular cAMP $[cAMP]_i$, both of which are required for CAD-induced lysosome-dependent death of MCF7 breast carcinoma and A549 non-small cell lung carcinoma cells.[19] To dissect the sequence of multiple CAD-induced intracellular events, we created HeLa cells expressing either a highly sensitive $[Ca^{2+}]_c$ indicator GCaMP6[33] or a $[cAMP]_i$ indicator Flamindo2, whose fluorescence intensity is reduced by cAMP binding.[34] In accordance with our previous data from other cell types,[19] the free $[Ca^{2+}]_c$ in HeLa cells was significantly increased already 5 min after the CAD treatment, whereas the increase in the $[cAMP]_i$ became significant 30 min after the treatment, simultaneously with the reduction in the pH_c (Figures 1A and 1B). Contrary to the relatively rapid changes in the free $[Ca^{2+}]_c$, $[cAMP]_I$, and pH_c, the lysosomal membrane permeabilization, as defined by the ability of a 15 kD protein galectin 1 (LGALS1) to enter the lysosomal lumen,[35] became detectable first 2–4 h after the addition of the drug (Figure 1E), while the integrity of the plasma membrane remained intact for additional 10 h (Figure S1A). A similar CAD-induced cytosolic acidification prior to lysosomal membrane permeabilization was also observed in A549 cells (Figures S1E and S1F).

These data indicate that CADs induce rapid and significant acidification of the cytosol that occurs shortly after the increase in the $[Ca^{2+}]_c$ but clearly before the lysosomal membrane permeabilization and cell death (Figure 1F).

CADs induce a rapid H^+ efflux from lysosomes

To enlighten the mechanism by which CADs cause the rapid cytosolic acidification demonstrated above, we first investigated the putative role of plasma membrane resident regulators of pH_c in this process. Most of them, including Na^+/H^+ exchanger 1 (NHE1/*SLC9A1*), Na^+/HCO_3^- co-transporters, and Na^+-driven Cl^-/HCO_3^- exchangers, are dependent on a high extracellular Na^+ concentration ($[Na^+]_e$ > 110 mM) to excrete cytosolic H^+. Accordingly, culturing HeLa-SypHer3s cells for 1 h in a low-Na^+-containing (40 mM) medium acidified the cytosol as efficiently as the treatment with CADs in a normal medium with 125 mM Na^+ (Figure 2A). In line with the dependence of NHE1-mediated H^+ export on high $[Na^+]_e$, the ability of ethyl isopropyl amiloride (EIPA), an inhibitor of NHE1,[36] to acidify the cytosol was abolished when cells were grown in low $[Na^+]_e$ (Figure 2B). In contrast, CAD antihistamines effectively acidified the cytosol in conditions where Na^+-dependent pH regulators in the plasma membrane were inhibited (Figure 2A). Akin to CADs, niclosamide, an H^+ carrier that dissipates H^+ from the endolysosomal compartment to the cytosol,[37,38] retained its ability to acidify the cytosol in low $[Na^+]_e$ (Figure 2A). Moreover, CAD antihistamines could further acidify the cytosol when NHE1 was specifically inhibited by EIPA (Figure 2C). To directly examine whether CADs have any effect on NHE1-mediated H^+ excretion, we performed an ammonium prepulse pH recovery assay. Contrary to EIPA, ebastine consistently failed to inhibit the pH recovery after NH_4Cl prepulse removal (Figure 2D). These data suggest that CADs have an experimentally negligible effect on NHE1 and other membrane-localized pH regulators dependent on high physiological sodium gradient across plasma membrane. Next, we tested whether CADs disrupted the H^+ gradient across the lysosomal membrane. Employing pH-sensitive (fluorescein isothiocyanate [FITC]) and pH-insensitive (tetramethylrhodamine [TMR]) dextrans as a pair of ratiometric sensors that reflect the relative lysosomal pH,[39] we demonstrated that terfenadine and ebastine significantly increased the lysosomal pH within 1 h (Figure 2E). Consistently, the fluorescence intensity of LysoTracker green that accumulates in lysosomes and other cytoplasmic acidic organelles was reduced significantly upon 1 h treatment with CADs (Figure 2F).

These data strongly suggest that the CAD-induced cytosolic acidification is a result of an early H^+ efflux from lysosomes with preserved membrane integrity and independent of Na^+-dependent pH regulators at the plasma membrane.

CAD-induced lysosomal H^+ efflux depends on lysosomal P2RX4 Ca^{2+} channel but not cAMP generation

To enlighten the regulation of the CAD-induced lysosomal H^+ efflux demonstrated above, we first examined the role of CAD-induced rapid Ca^{2+} release in this process. Interestingly, small interfering RNA (siRNA)-mediated depletion of *P2RX4*, a Ca^{2+} channel previously demonstrated to mediate the CAD-induced lysosomal Ca^{2+} release,[19] effectively blocked CAD-induced cytosolic acidification in SypHer3s-expressing HeLa and MCF7 cells (Figures 3A and S2A). In contrast, siRNA-mediated depletion of P2X7 (*P2RX7*), a purinergic receptor on the plasma membrane, had no effect on CAD-induced cytosolic acidification (Figure S2B).

In MCF7 cells, the CAD-induced P2RX4-dependent lysosomal Ca^{2+} release activates the generation of cAMP by the Ca^{2+}/calmodulin-dependent adenyl cyclase 1 (ADCY1).[19] However, the siRNA-mediated depletion of the Ca^{2+}/ADCY1, which effectively reduced the $[cAMP]_i$ (Figure 3B), had no effect on CAD-induced cytosolic acidification in MCF7 cells (Figure 3C). In line with this, the strong induction of cAMP generation by forskolin failed to acidify the cytosol and to enhance terfenadine-induced cytosolic acidification in HeLa cells (Figures 3D and S2C).

Based on the above, we conclude that the CAD-induced lysosomal H^+ efflux depends on the P2RX4-mediated lysosomal Ca^{2+} release but is independent of CAD-induced cAMP signaling pathways.

CAD-induced cytosolic acidification inhibits the transcriptional activity of STAT3

Cytosolic acidification has proven to be a powerful means to inhibit the activity of the oncogenic transcription factor STAT3.[12] It triggers the dephosphorylation of Y705-STAT3 and the subsequent translocation of STAT3 from the nucleus to the lysosomal membrane, where STAT3 assists the V-ATPase to pump excessive cytosolic H^+ to the lysosomal lumen. Inspired by the ability of CADs to induce rapid cytosolic acidification in

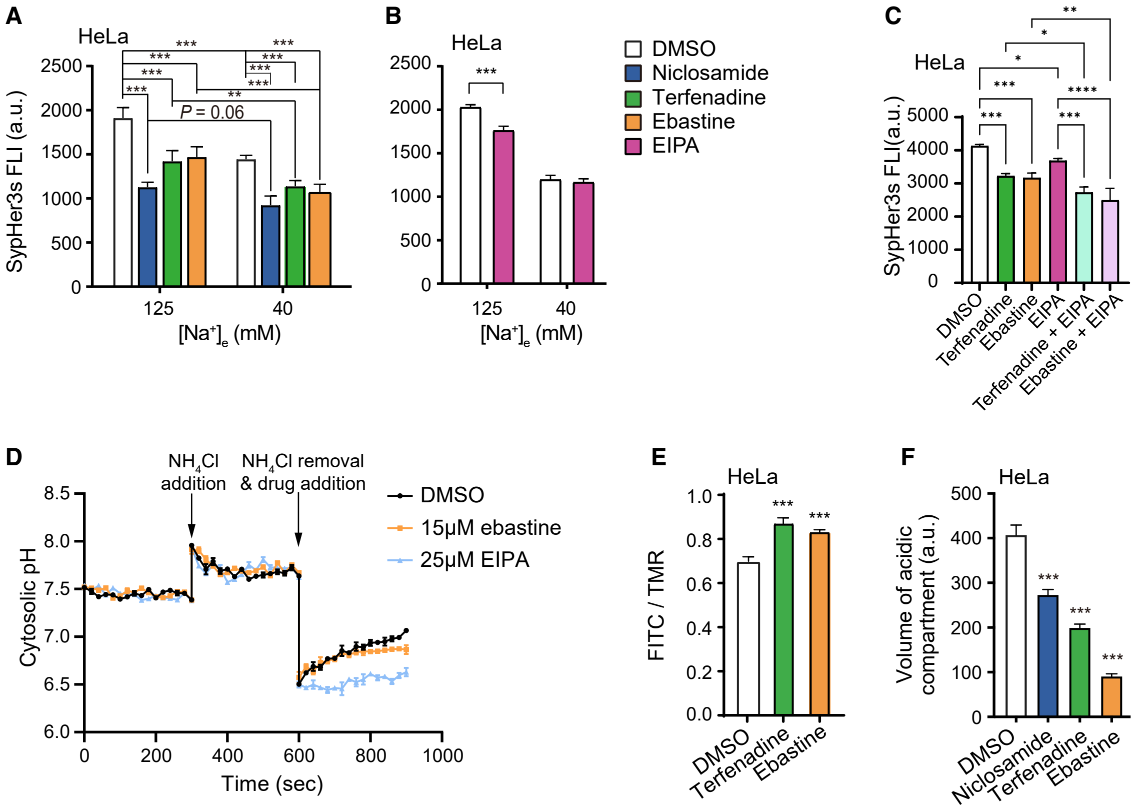

Figure 2. CADs induce an early efflux of lysosomal H^+

(A and B) SypHer3s FLI in HeLa-SypHer3s cells treated for 1 h with DMSO, 10 μM niclosamide (positive control for acidification), 6 μM terfenadine, or 15 μM ebastine (A) or for 4 h with 25 μM EIPA (positive control for Na^+ dependent acidification) with normal (125 mM) or low (40 mM) $[Na^+]$ in the media (B) and analyzed by flow cytometry.

(C) SypHer3s FLI in HeLa-SypHer3s cells treated for 2 h with DMSO, 6 μM terfenadine, 15 μM ebastine, 25 μM EIPA, or 6 μM terfenadine + 25 μM EIPA or 15 μM ebastine + 25 μM EIPA in live-cell imaging solution and analyzed by flow cytometry.

(D) Real-time pH monitoring during ammonium prepulse assay. HeLa cells stained by BCECF-AM were treated as indicated. Upon the removal of the NH_4Cl prepulse, the cytosol underwent a pH recovery in DMSO and ebastine-treated cells but not EIPA (NHE1 inhibitor)-treated cells.

(E) Mean ratios of FLIs of lysosomal pH-sensitive (FITC; fluorescence reduced by low pH) and pH-insensitive (TMR) dextran probes in HeLa cells treated for 1 h with DMSO, 6 μM terfenadine, or 15 μM ebastine and analyzed by confocal microscopy and ImageJ software.

(F) Relative volumes of acidic compartments in HeLa cells treated as in (A) (in normal medium), stained with Lysotracker green, and analyzed by flow cytometry. Error bars, SD of three independent experiments with 10,000 (A–C and F) or 10 (E) randomly chosen cells analyzed in each experiment. *$p < 0.05$; **$p < 0.01$; ***$p < 0.001$ as analyzed by one-way ANOVA (C, E, and F) or two-way ANOVA (A and B) with Holm-Sidak (C), Tukey (E and F), or Dunnett (A and B) for multiple group comparison.

cancer cells, we tested whether CADs could serve as STAT3 inhibitors. To visualize the subcellular localization of STAT3, we employed A549 triple cells, in which the NH_2 terminus of the endogenous STAT3 gene is fused to a red fluorescent protein (RFP).[40] Treatment of these cells with terfenadine or ebastine for 2 or 4 h resulted in a significant increase in the colocalization of RFP-STAT3 with LAMP1-BFP-labeled lysosomes (Figures 4A, 4B, and S3), while nuclear RFP-STAT3 was reduced by 2 h ebastine treatment (Figure 4C), suggestive of CAD-induced inhibition of the transcriptional activity of STAT3. Thus, we investigated the effect of CADs on the phosphorylation of Y705-STAT3 in HeLa, A549 cells, and PANC-1 (pancreatic ductal adenocarcinoma) cells, all of which are characterized by the hyperactivation of STAT3.[41–43] Treatment of HeLa cells with four clinically relevant CADs, terfenadine, ebastine, astemizole, or penfluridol, at concentrations that kill approximately 40% of the cells in 24 h, inhibited 40%–55% of the phosphorylation of Y705-STAT3 already after a 30 min treatment, and the inhibition persisted throughout the 16 h followup (Figures 5A and 5B, top). This inhibitory effect was followed by a significant reduction in the expression of cyclin D1, a growth-promoting protein encoded by a STAT3 target gene *CCND1* (Figures 5A and 5B, bottom). CADs caused only minor changes in the expression of STAT3 or its phosphorylation at the serine 727 (S727) residue (Figures 5A and S4A), which has been implicated in non-canonical, mitochondrial functions of STAT3.[44,45] Importantly, the inhibitory effects of CADs on the phosphorylation of Y705-STAT3 and its transcriptional activity were not limited to HeLa cells but were even stronger in A549 and PANC-1 cells (Figures S4B–S4E). Furthermore, all CADs showed similar or even more potent

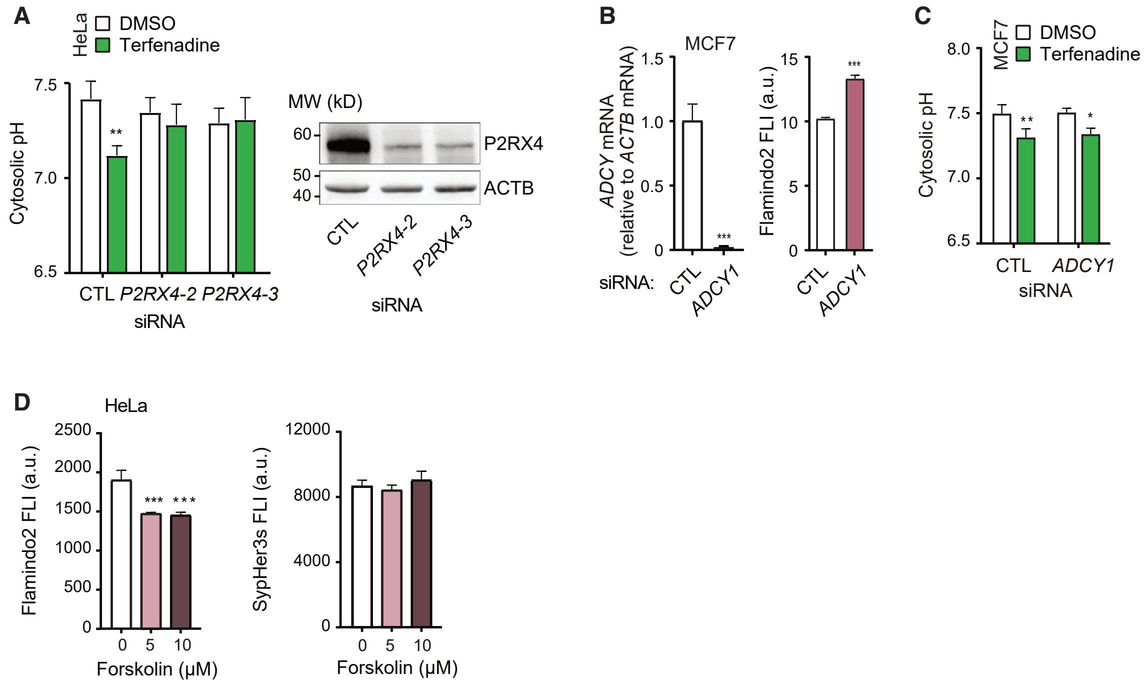

Figure 3. CAD-induced cytosolic acidification depends on P2RX4-mediated lysosomal Ca^{2+} release but not cAMP generation

(A) Cytosolic pH values of HeLa-SypHer3S cells treated with indicated siRNAs for 72 h and with either DMSO or 6 μM terfenadine for the last 2 h (left) were defined as in Figure 1C. Representative (n = 3) immunoblots of indicated proteins in siRNA-treated HeLa-SypHer3S cells (right).

(B) Relative ADCY1 mRNA levels (left) and Flamindo2 FLI (right) in MCF7-Flamindo2 cells treated with indicated siRNAs for 72 h.

(C) Cytosolic pH values of MCF7-SypHer3s cells treated with indicated siRNAs for 72 h and with either DMSO or 6 μM terfenadine for the last 2 h were analyzed as in Figure 1C.

(D) Flamindo (left) and SypHer3s (right) FLIs in HeLa cells treated with indicated concentrations of forskolin for 1 h and analyzed by flow cytometry.

Error bars, SD of three independent experiments with ≥10 (A and C) or ≥10,000 cells (D) randomly chosen cells analyzed in each sample. *$p < 0.05$; **$p < 0.01$; ***$p < 0.001$ as analyzed by one-way ANOVA (B and D) or two-way ANOVA (A and C) with Tukey (B and D) or Dunnett (A and C) multiple comparison.

inhibition of Y705-STAT3 phosphorylation and *CCND1* expression than conventional STAT3 inhibitors, WP1066,[46] STA-21,[47] and stattic,[48] applied at their recommended concentrations (Figures 5C and 5D). These small molecules have been reported to inhibit the phosphorylation (WP1066 and stattic) or dimerization (STA-21) of STAT3. Notably, none of them were able to induce cytosolic acidification (Figure 5E), which is the likely mechanism of CAD-mediated STAT3 dephosphorylation. Supporting this mechanism, the siRNA-mediated depletion of the upstream effectors of CAD-induced lysosomal H^+ efflux, *P2RX4*, significantly reduced the terfenadine-induced dephosphorylation of Y705-STAT3, demonstrated by the changes of both the extent and amount of Y705-phosphorylated STAT3 (Figures 5F, 5G, and S4F).

To study the kinetics and dose responses of CAD-induced STAT3 inhibition, we employed the A549 triple cell line with a secreted luciferase inserted downstream of the endogenous *CCND1* promoter.[40] In line with the rapid CAD-induced dephosphorylation of P-Y705-STAT3 (Figures 5A, 5B, and S4B–S4E), terfenadine and ebastine inhibited the activity of the STAT3-responsive *CCND1* promoter significantly already after 1 h treatment (Figure 6A). The half-maximal inhibitory concentrations (IC_{50}) of 2 h treatments with terfenadine, ebastine, and WP1066 were 7.67, 15.82, and 7.90, respectively (Figure 6B). Notably, the maximal inhibitory capacity of WP1066 was only around 55%, whereas higher concentrations of terfenadine and ebastine inhibited up to 80% of the *CCND1* promoter activity (Figure 6B). In line with the effective inhibition of the *CCND1* promoter in A549 triple cells, treatment of HeLa cells with terfenadine and ebastine for 2 h significantly reduced the level of *CCND1* mRNA (Figure 6C). To enlighten the overall effect of CADs on reported STAT3-regulated genes,[49] we performed whole-transcriptome sequencing of CAD-treated HeLa cells. Based on three independent RNA sequencing (RNA-seq) datasets, 2 h treatment with terfenadine and ebastine significantly downregulated 136 and 159 STAT3 target genes, respectively, of which 113 were common to both CADs (Figure 6D; Tables S2 and S3). Prolonging the treatment time to 8 h increased the number of downregulated STAT3 target genes to 214 for terfenadine and 235 for ebastine, of which 139 were common to both CADs (Figure 6D; Tables S2 and S3). In addition to *CCND1*, several other cancer-relevant genes, including *MCL1*, *BCL2L1*, *CDC25*, *FAS*, and *HIF1A*, were among the common genes (Figures S5A and S5B). Furthermore, we compared the transcriptomic changes in CAD-treated HeLa cells and HeLa STAT3 knockout (KO) cells. Intriguingly, there were 173 and 186 STAT3 target genes specifically downregulated by terfenadine and ebastine, respectively. KEGG pathway enrichment

CellPress
OPEN ACCESS

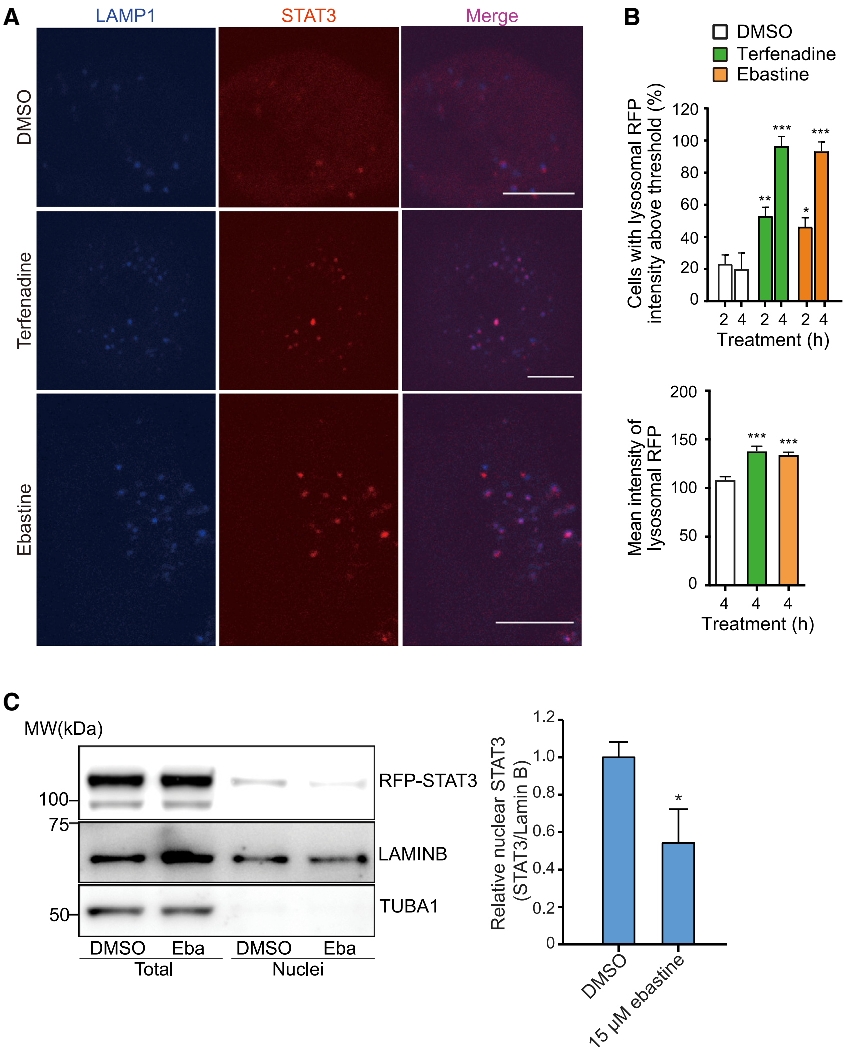

Figure 4. CADs trigger lysosomal translocation of STAT3

(A) Representative images of A549 triple cells with RFP-tagged endogenous STAT3 transfected with LAMP1-BFP and treated with DMSO, 6 μM terfenadine, or 15 μM ebastine for 4 h. Scale bars, 10 μm. See Figure S3 for corresponding images of cells treated for 2 h.

(B) Percentage of cells treated as in (A) with the average intensity of lysosomal RFP-STAT3 above a selected threshold (top) and mean intensities of lysosomal RFP-STAT3 (bottom) in cells treated as in (A).

(C) Representative immunoblots of indicated proteins in A549 triple cells treated with 15 μM ebastine for 2 h (left) and relative quantification of nuclear STAT3 (right).

Error bars, SD of three independent experiments with 10 randomly chosen cells analyzed in each sample. *$p < 0.05$; **$p < 0.01$; ****$p < 0.0001$ as analyzed by one-way ANOVA with Tukey multiple group comparison.

analysis showed that these two groups of STAT3 target genes are mostly implicated in cancer-promoting pathways, such as PI3K-Akt signaling, focal adhesion, Ras signaling, and p53 signaling (Figures S5C and S5D). As STAT3 is also an essential transcription factor for many normal physiological processes, this unique feature may empower CADs to inhibit STAT3 in a more cancer-specific manner.

The data presented above introduce commonly used CADs as effective inhibitors of the Y705 phosphorylation, nuclear localization, and transcriptional activity of STAT3 likely due to their ability to trigger lysosomal H^+ efflux and rapid cytosolic acidification.

CAD-induced cytosolic acidification sensitizes cancer cells to STAT3 inhibition

Lastly, we sought to investigate whether CAD-induced cytosolic acidification without the following lysosome membrane permeabilization (LMP) holds any anticancer activity. The ability of low, non-lethal concentrations of terfenadine and ebastine to induce cytosolic acidification without disrupting lysosomal membrane integrity (Figures 7A and 7B) allowed us to examine the effect of CAD-induced lysosomal H^+ efflux on the survival of cells depleted of STAT3. Noteworthy, these non-lethal concentrations of CADs retained their effective STAT3-inhibitory effect (Figures S6A and S6B). As we have reported previously,[12] the KO of STAT3 in HeLa cells lowered the pH_c from approximately 7.5 to below 7.3 without compromising cell survival (Figures 7C and 7D, left). The treatment of HeLa-STAT3-KO cells with 4 μM terfenadine lowered the pH_c further to 7.07 (KO-1) or 6.78 (KO-11) already after 30 min and resulted in a significant increase in cell death after 24 h (Figure 7D). A partial siRNA-mediated depletion or STA21-mediated pharmacological inhibition of STAT3 combined with terfenadine in HeLa cells (Figures 7C, 7E, and S6C) as well as the combination of WP1066 with either terfenadine or ebastine in both HeLa and A549 cells had similar synergistic effects (Figures 7F, left and middle, and S6D). Statistically significant synergistic effects of the treatments were confirmed by determining synergy scores by the Bliss independent model.[50] It should be noted that unlike the loss of STAT3, which acidified the cytosol per se and, together with terfenadine treatment, resulted in an excessive pH drop, STAT3 inhibition by WP1066 had no effect on pH_c (Figures 5E and S6E) but still caused synergistic cell death together with 4 μM terfenadine treatment. This strongly suggests that CAD-induced cytosolic acidification sensitizes cancer cells to STAT3 inhibition-induced cell death. Supporting this conclusion, EIPA, which acidifies the cytosol by inhibiting the NHE1-mediated proton extrusion at the plasma membrane, also strongly sensitized HeLa cells to WP1066-induced cell death (Figure 7F, right).

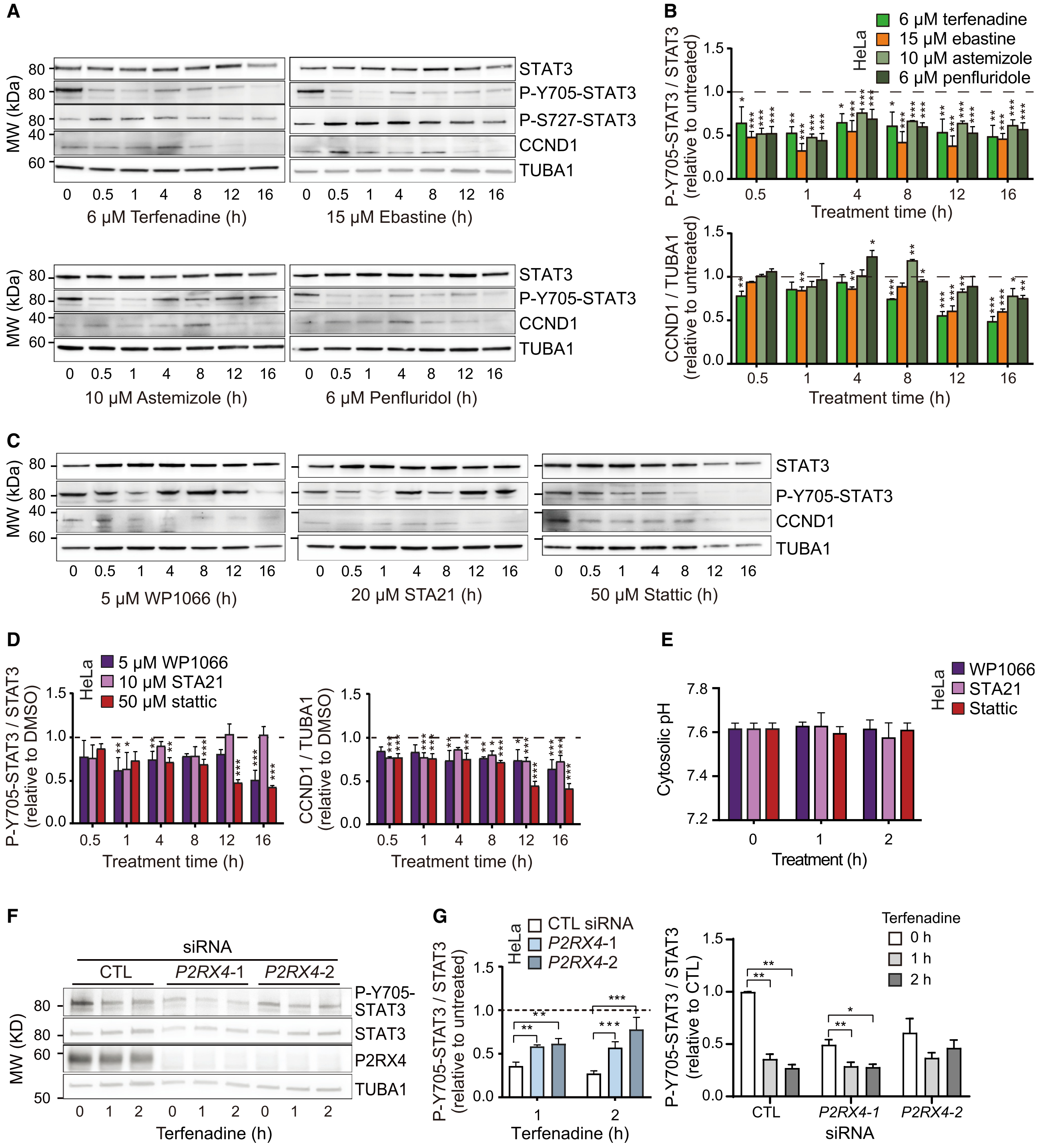

Figure 5. CADs reduce Y705-STAT3 phosphorylation and CCND1 expression

(A–D) Representative immunoblots of indicated proteins in HeLa cells treated with indicated CADs (A) or STAT3 inhibitors (C) for 0–16 h and the quantification of blots from three independent experiments (B and D). See Figure S3 for the quantification of P-S727-STAT3 and total STAT3 (Figure S3A) as well as similar data from other cancer cells (Figure S3B–S3E).

(E) Cytosolic pH values of HeLa-SypHer3s cells left untreated or treated with 5 μM WP1066, 10 μM STA21, or 50 μM stattic for indicated time and analyzed as in Figure 1C.

(F) Representative immunoblots of indicated proteins in HeLa cells transfected with *P2RX4* siRNAs for 72 h. When indicated, cells were treated with 6 μM terfenadine for the last 1 or 2 h.

(legend continued on next page)

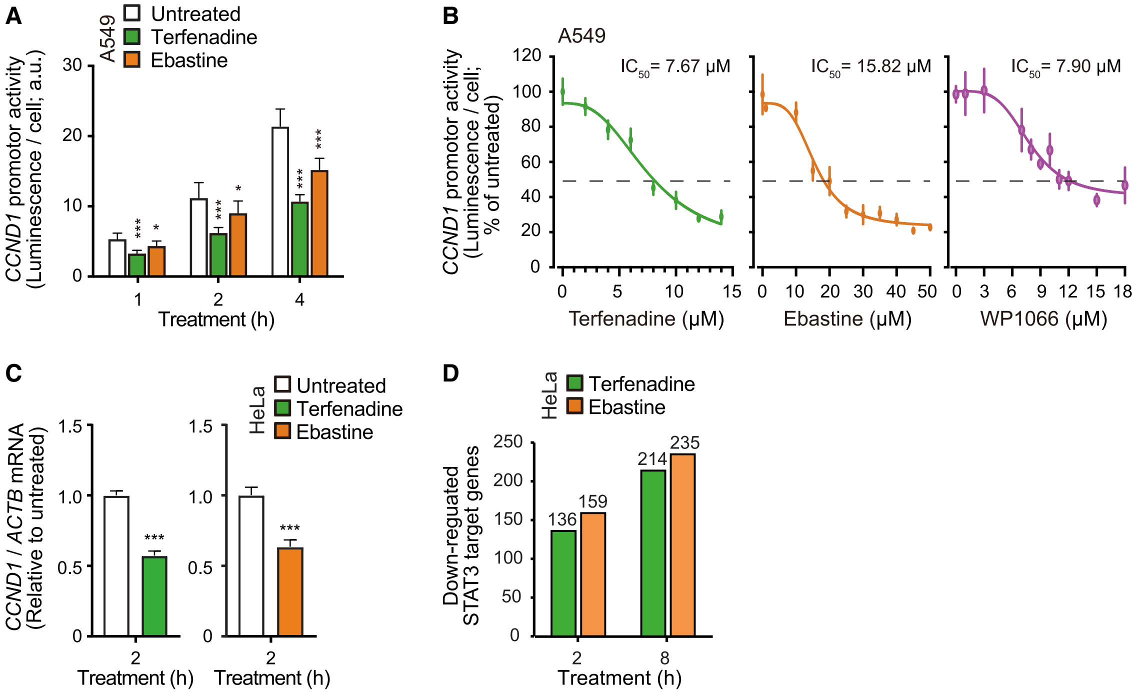

Figure 6. CADs inhibit the transcriptional activity of STAT3

(A) Luciferase activity in the media of A549 triple cells expressing *CCND1* promoter-driven secreted luciferase. Cells were left untreated or treated with 6 μM terfenadine or 15 μM ebastine for the indicated times.

(B) Luciferase activity in the media of A549 triple cells treated with indicated concentrations of terfenadine, ebastine, or WP1066 for 2 h. IC_{50}, half-maximal inhibitory concentration.

(C) Relative *CCND1* mRNA levels in HeLa cells left untreated or treated with 6 μM terfenadine or 15 μM ebastine for 2 h and analyzed by qPCR. *ACTB* mRNA served as an internal control.

(D) Number of downregulated STAT3 target genes (fold change > 1.5 and adjusted $p < 0.05$) in HeLa cells treated as in (C) for 2 and 8 h and analyzed by RNA-seq. See Figure S5 for a more detailed transcriptomic analysis.

Error bars, SD of three independent experiments. *$p < 0.05$; **$p < 0.01$; ***$p < 0.001$ as analyzed by one-way ANOVA (C) or two-way ANOVA (A) with Tukey (C) or Dunnett (A) multiple comparison.

Contrary to the lysosome-dependent cell death induced by lethal concentrations of CADs, the combination of sublethal concentrations of ebastine and WP1066 did not induce lysosomal membrane permeabilization detectable by LGALS1 puncta assay (Figure 7G). Instead, it induced the intrinsic apoptosis pathway as indicated by a significant increase in mitochondrial outer membrane permeabilization, which was visualized by the formation of BAX puncta (Figure 7H). In line with this, N-benzyloxycarbonyl-Val-Ala-Asp(O-Me) fluoromethyl ketone (zVAD-fmk), when used at concentrations that effectively inhibit apoptotic caspases but not lysosomal cysteine cathepsins,[51] provided almost complete protection against the combination treatment (Figure 7I). Notably, necrostatin-1, an inhibitor of necroptosis-inducing, receptor-interacting Ser/threonine-protein kinase 1,[52] provided partial but significant protection, while ferrostatin-1, which inhibits ferroptosis by trapping peroxyl radicals,[53] had no protective effect (Figure 7I).

Finally, we examined the effect of the combination treatment on subcutaneous A549 lung cancer xenografts in female Balb/c *nude* mice. While thrice-weekly treatment with neither 30 mg/kg ebastine nor 20 mg/kg WP1066 influenced tumor growth, the tumors in mice treated with the combination of the two treatments grew significantly slower than tumors in the vehicle-, WP1066-, or ebastine-treated mice (Figure 7J).

Collectively, these results highlight the antitumor potential of CAD-induced lysosomal H^+ efflux and cytosolic acidification and provide a novel drug combination strategy for cancer treatment.

DISCUSSION

Cancer progression is associated with enlargement and aberrant activation of the lysosomal compartment.[4] Such lysosomal changes do not only support the demanding cancer metabolism

(G) The quantification of blots from three independent experiments performed as (F). The residue P-Y705-STAT3 in terfenadine-treated cells relative to untreated cells is displayed in the left panel. The overall relative P-Y705-STAT3 in all samples is displayed in the right panel.

Error bars, SD of three independent experiments (A–D) or each with 10 randomly chosen cells analyzed in each sample (E). *$p < 0.05$; **$p < 0.01$; ***$p < 0.001$ as analyzed by one-way ANOVA (E) or two-way ANOVA (A–D and G) with Tukey (E) or Dunnett (A–D and G) multiple comparison.

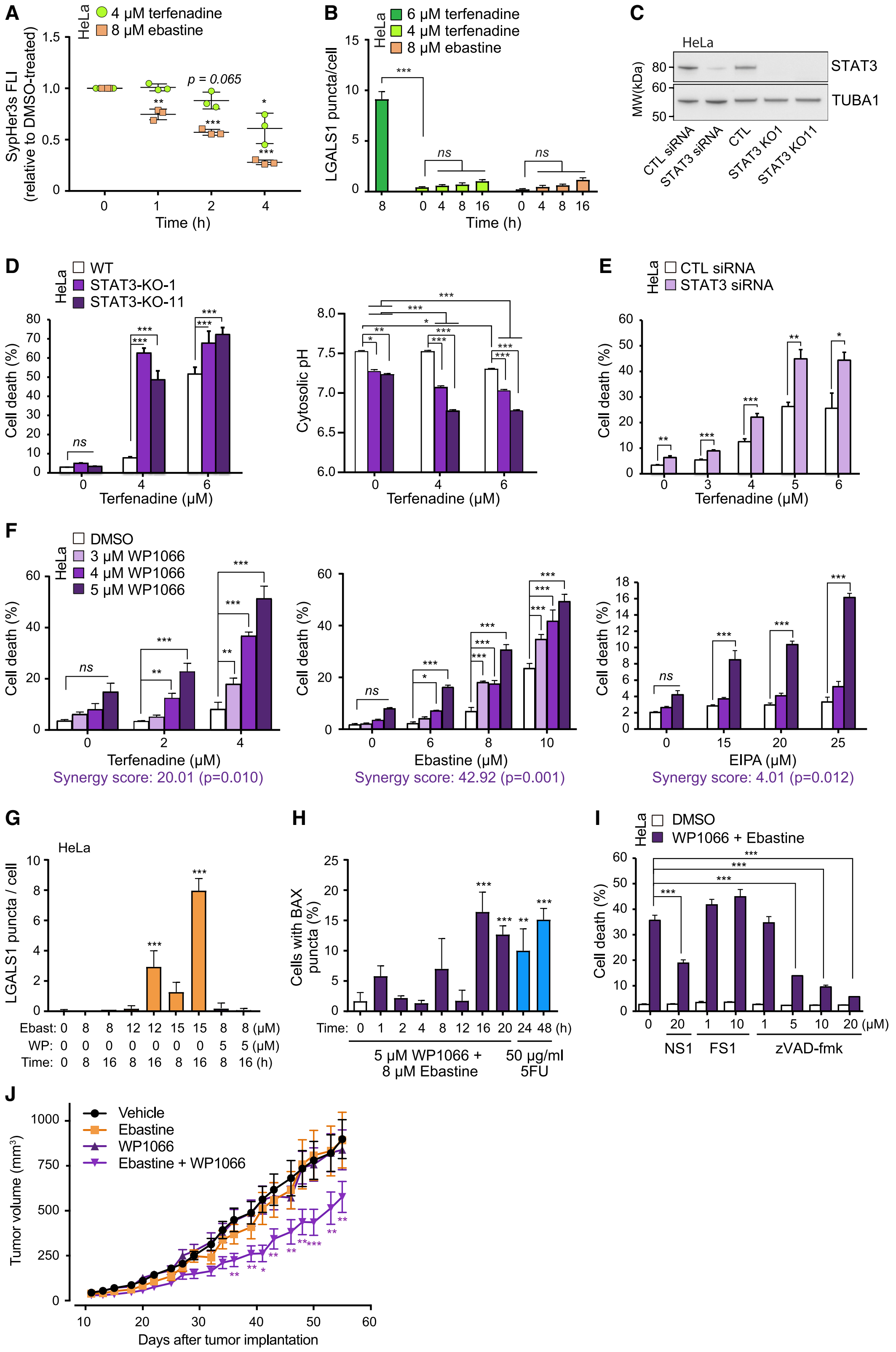

(legend on next page)

CellPress
OPEN ACCESS

but contribute also to the invasiveness and metastatic potential of cancer cells.[54,55] Accordingly, commonly used cationic amphiphilic antihistamines and other CADs that disturb lysosomal function and induce cancer-specific lysosome-dependent cell death are emerging as potential candidates for cancer treatment.[4] The data presented above introduce rapid cytosolic acidification as a novel anticancer function of CADs. Because lysosomes constitute a significant reservoir of the intracellular H^+, cytosolic acidification is an obvious consequence of the loss of lysosomal membrane integrity. The present study shows, however, that CAD-induced cytosolic acidification occurs clearly before lysosomal membranes become permeable to small proteins and that it can be triggered by low CAD concentrations that induce neither lysosomal membrane permeabilization nor cell death. Supporting lysosomal H^+ efflux as the cause of the CAD-induced cytosolic acidification, the decrease in pH_c coincided with an increase in lysosomal pH and was not affected by the inhibition of Na^+-dependent pH regulators in the plasma membrane. Whereas the CAD-induced loss of lysosomal membrane integrity depends on the P2RX4-mediated lysosomal Ca^{2+} release and the subsequent ADCY1-dependent increase in intracellular cAMP,[19] siRNA-based experiments demonstrated the dependence of the CAD-induced cytosolic acidification solely on *P2RX4* but not on *ADCY1*. These data identify two divergent events initiated by the lysosomal Ca^{2+} release: one leading to the cAMP generation, loss of lysosomal membrane integrity, and cell death and the other triggering lysosomal H^+ efflux and cytosolic acidification. While the molecular basis of the CAD-induced lysosomal H^+ efflux remains unknown, its dependence on Ca^{2+} suggests an involvement of a yet unidentified lysosomal Ca^{2+}/H^+ exchanger. Given that the lysosomal Ca^{2+}/H^+ exchanger and Ca^{2+} refilling from the endoplasmic reticulum (ER) are proposed to establish the lysosomal Ca^{2+} gradient, the present study provides an experimental framework that will potentially lead to the identification of the enigmatic lysosomal Ca^{2+}/H^+ exchanger and the validation of the two theories.

STAT3 is an oncogenic transcription factor that is hyperphosphorylated on its Y705 residue and transcriptionally activated in around 70% solid and hematological cancers.[56] Because of its essential role in promoting tumorigenesis, STAT3 has been considered as an attractive drug target for more than 20 years.[57] Yet, the efficacy of existing STAT3 inhibitors, which either inhibit the Y705 phosphorylation or disrupt the interaction between STAT3 and DNA, has been disappointing, and none of them have been approved for clinical use. We have previously demonstrated that cytosolic acidification induces the translocation of STAT3 to lysosomal membranes, thereby inhibiting the transcriptional activity of STAT3 in the nucleus.[12] In line with this, our new data show that CAD-induced cytosolic acidification is associated with effective inhibition of Y705-STAT3 phosphorylation, translocation of STAT3 to lysosomes, and inhibition of STAT3 transcriptional activity. This inhibition is likely to be due to CAD-induced Ca cytosolic acidification, as it can be inhibited by the depletion of *P2RX4*. Thus, CAD-induced cytosolic acidification emerges as an effective way to inhibit STAT3 in cancer cells and may partially explain the observed anticancer effects of CADs *in vivo*.[4] However, the molecular mechanism of how cytosolic acidification induces STAT3 dephosphorylation remains to be investigated. One possibility is that an unknown STAT3 phosphatase is activated when pH_c is lowered to a certain threshold. Alternatively, due to STAT3 translocation induced by the cytosolic acidification, exposure of nuclear STAT3 to its cytosolic phosphatases might trigger STAT3 dephosphorylation. Both possibilities will be experimentally assessed in future studies.

CADs affect STAT3 target gene expressions with clearly different kinetics. Similar differences in kinetics have been reported previously upon both genetic and pharmacological inhibition of STAT3 inhibitors.[58,59] The most likely explanation for the varied kinetics is that many of the STAT3 target genes are essential for cell cycle, cell survival, apoptosis, and metabolism, and they are tightly regulated by a cohort of signaling pathways and transcription factors. Thus, the kinetics of their expression is not solely regulated by STAT3 but is dependent on the interconnected and coordinated regulatory network; for example, MCL1 is regulated at least by ATF4, STAT3, CREB, and c-Myc.[60]

In addition to the rewired lysosomes, cancer cells are addicted to the alkaline cytosol (pH > 7.2), which enables tumor

Figure 7. CAD-induced cytosolic acidification exerts a synergistic antitumor effect with WP1066

(A) SypHer3s FLI in HeLa cells treated with 4 μM terfenadine or 8 μM ebastine for indicated times.

(B) Number of $LGALS1^+$ leaky lysosomes in HeLa cells treated with terfenadine or ebastine as indicated.

(C) Representative immunoblots of STAT3 and TUBA (loading control) in lysates of HeLa cells treated with indicated siRNAs for 72 h and indicated Hela-STAT3-KO clones (n > 3).

(D) Death (left) and cytosolic pH (right) of indicated HeLa cell clones treated with terfenadine for 24 h (left) or 30 min (right). Cells were stained with propidium iodide (dead cells) and Hoechst-33342 (total cells), and cell death was analyzed by Celigo Imaging Cytometer. Cytosolic pH was determined by pHrodo-AM (see STAR Methods).

(E) Death of HeLa cells treated with indicated siRNAs for 72 h and with indicated concentrations of terfenadine for the last 24 h. Cell death was analyzed as in (D).

(F) Death of HeLa cells treated as indicated with WP1066 together with terfenadine (left), ebastine (middle), or EIPA (right) for 24 h. Cell death was analyzed as in (D). Synergy score was determined by the Bliss independent model (see STAR Methods).

(G) Number of $LGALS1^+$ leaky lysosomes in HeLa cells treated with ebastine and WP1066 as indicated.

(H) Percentage of $BAX\ puncta^+$ apoptotic HeLa cells after indicated treatments. 5FU served as a positive control for apoptosis.

(I) Death of HeLa cells treated with DMSO or 5 μM WP1066 + 8 μM ebastine together with indicated concentrations of necrostatin 1 (NS1), ferrostatin 1 (FS1), or zVAD-FMK for 24 h. Cell death was analyzed as in (D).

(J) Growth of subcutaneous A549 xenografts in nude female BALB/c mice treated with vehicle, 30 mg/kg ebastine, 20 mg/kg WP1066, or a combination of the two drugs 3 times a week.

Error bars, SD of three independent experiments with ≥10 randomly chosen cells (B, D, right, G, and H) or ≥1,000 cells (A, D, left, E, F, and I) analyzed in each sample. SD of ≥15 animals is displayed in the xenograft experiments. *$p < 0.05$; **$p < 0.01$; ***$p < 0.001$ as analyzed by one-way ANOVA with Tukey multiple comparison (A, B, and G–I) or two-way ANOVA with Dunnett multiple comparison (D–F and J).

progression by promoting proliferation, survival, metabolic adaption, cell migration, and invasion.[11] To cope with the continuous acid stress caused by an overload of H^+ produced by aerobic glycolysis, cancer cells spend a lot of energy removing H^+ from the cytosol.[6,11] Thus, the ability of CADs to enhance the cellular acid stress even at relatively low concentrations may prove to be essential for their multiple anticancer effects observed *in vivo*.[4] Here, we demonstrate synergistic cytotoxicity by combining CADs with the inhibition of STAT3. This combination treatment results in caspase-dependent apoptotic cell death, which is in line with the reported ability of low pH to promote caspase activation.[61] It remains to be studied whether CADs also sensitize cancer cells to other apoptosis-inducing drugs and how the combination treatment triggers the apoptosis. In any case, the increased lysosomal pH induced by the H^+ efflux is likely to contribute to the previously described ability of CADs to revert multidrug resistance by reducing the trapping of basic cancer drugs inside the acidic lysosomes.[20,28] The synergistic anticancer activity by the combination of CADs and WP1066 in a murine xenograft mode supports the significance of CAD-induced lysosomal H^+ leak *in vivo*. As H^+ flows dynamically en route of membrane trafficking and can be eventually exocytosed, while lysosome maturation and reformation constantly replenish lysosome luminal H^+, we postulate that CAD-induced lysosomal H^+ leak may be a continuous pH regulatory event *in vivo*. As lysosomes are dispersed inside cells, it is conceivable that membrane-localized transporters are not able to resolve continuous H^+ leak from lysosomes in time. To experimentally validate this hypothesis, a trustworthy method to measure pH_c in tumors needs to be established.

In conclusion, the data presented above identify an induced lysosomal H^+ efflux, cytosolic acidification, and STAT3 inhibition as novel anticancer mechanisms of CADs. The ability of CADs to potently inhibit the transcriptional activity of STAT3 is of particular interest because the strong clinical applicability and the repurposing potential of cationic amphiphilic antihistamines for cancer therapy might circumvent the dilemma that none of the conventional STAT3 inhibitors have passed stringent clinical trials.

Limitations of the study

Animal studies were performed without consideration of gender equality. Validations in male mice should be done before any further translational studies.

STAR★METHODS

Detailed methods are provided in the online version of this paper and include the following:

- KEY RESOURCES TABLE
- RESOURCE AVAILABILITY
  - Lead contact
  - Materials availability
  - Data and code availability
- EXPERIMENTAL AND SUBJECT DETAILS
  - Cell lines
  - Animals
- METHOD DETAILS
  - Transfections
  - siRNA
  - Western blot
  - Immunostaining
  - Imaging
  - Real-time PCR
  - Fluo 4 staining
  - Luciferase assay
  - Lysosomal pH measurement
  - Cytosolic pH measurement
  - Ammonium prepulse assay
  - Nuclear fractionation
  - Cell death
  - Synergy score determination
  - RNA seq
  - Xenografts
- QUANTIFICATION AND STATISTICAL ANALYSIS

SUPPLEMENTAL INFORMATION

Supplemental information can be found online at https://doi.org/10.1016/j.celrep.2023.112137.

ACKNOWLEDGMENTS

We thank Dr. Dmitry Malkov for providing valuable reagents and Louise Vanderfox and Tiina Naumanen Dietrich for technical assistance. The research reported in this publication was supported by European Research Council (AdG 340751 to M.J.), Danish National Research Foundation (DNRF125 to M.J.), Danish Cancer Society (R167-A11061 and R269-A15695 to M.J.), Danish Council for Independent Research (DFF-7016-00360 to M.J.), and Novo Nordisk Foundation (NNF15OC0016914 to M.J.).

AUTHOR CONTRIBUTIONS

M.J. and B.L. designed the research, analyzed the results, and wrote the manuscript. B.L. and R.C. performed most of the cell biology, biochemical, and molecular experiments. C.Z. supervised and Y.Z. performed the mouse study. J.H. and Y.L. conducted the RNA seq study.

DECLARATION OF INTERESTS

The authors declare no competing interests.

Received: June 15, 2022
Revised: November 8, 2022
Accepted: February 2, 2023
Published: February 17, 2023

REFERENCES

1. Luo, J., Solimini, N.L., and Elledge, S.J. (2009). Principles of cancer therapy: oncogene and non-oncogene addiction. Cell *136*, 823–837. https://doi.org/10.1016/j.cell.2009.02.024.
2. Nagel, R., Semenova, E.A., and Berns, A. (2016). Drugging the addict: non-oncogene addiction as a target for cancer therapy. EMBO Rep. *17*, 1516–1531. https://doi.org/10.15252/embr.201643030.
3. Condon, K.J., and Sabatini, D.M. (2019). Nutrient regulation of mTORC1 at a glance. J. Cell Sci. *132*, jcs222570. https://doi.org/10.1242/jcs.222570.
4. Ellegaard, A.M., Bach, P., and Jäättelä, M. (2023). Targeting cancer lysosomes with good old cationic amphiphilic drugs. Rev. Physiol. Biochem. Pharmacol. *185*, 107–152. https://doi.org/10.1007/112_2020_56.

5. Jacobs, K.A., Maghe, C., and Gavard, J. (2020). Lysosomes in glioblastoma: pump up the volume. Cell Cycle *19*, 2094–2104. https://doi.org/10.1080/15384101.2020.1796016.

6. Chen, R., Jäättelä, M., and Liu, B. (2020). Lysosome as a central hub for rewiring PH homeostasis in tumors. Cancers *12*, 2437. https://doi.org/10.3390/cancers12092437.

7. Xu, H., and Ren, D. (2015). Lysosomal physiology. Annu. Rev. Physiol. *77*, 57–80. https://doi.org/10.1146/annurev-physiol-021014-071649.

8. Li, P., Gu, M., and Xu, H. (2019). Lysosomal ion channels as decoders of cellular signals. Trends Biochem. Sci. *44*, 110–124. https://doi.org/10.1016/j.tibs.2018.10.006.

9. Sterea, A.M., Almasi, S., and El Hiani, Y. (2018). The hidden potential of lysosomal ion channels: a new era of oncogenes. Cell Calcium *72*, 91–103. https://doi.org/10.1016/j.ceca.2018.02.006.

10. Jefferies, K.C., Cipriano, D.J., and Forgac, M. (2008). Function, structure and regulation of the vacuolar (H+)-ATPases. Arch. Biochem. Biophys. *476*, 33–42. https://doi.org/10.1016/j.abb.2008.03.025.

11. Webb, B.A., Chimenti, M., Jacobson, M.P., and Barber, D.L. (2011). Dysregulated pH: a perfect storm for cancer progression. Nat. Rev. Cancer *11*, 671–677. https://doi.org/10.1038/nrc3110.

12. Liu, B., Palmfeldt, J., Lin, L., Colaço, A., Clemmensen, K.K.B., Huang, J., Xu, F., Liu, X., Maeda, K., Luo, Y., and Jäättelä, M. (2018). STAT3 associates with vacuolar H(+)-ATPase and regulates cytosolic and lysosomal pH. Cell Res. *28*, 996–1012. https://doi.org/10.1038/s41422-018-0080-0.

13. Demaurex, N. (2002). pH Homeostasis of cellular organelles. News Physiol. Sci. *17*, 1–5. https://doi.org/10.1152/physiologyonline.2002.17.1.1.

14. Paroutis, P., Touret, N., and Grinstein, S. (2004). The pH of the secretory pathway: measurement, determinants, and regulation. Physiology *19*, 207–215. https://doi.org/10.1152/physiol.00005.2004.

15. Cang, C., Bekele, B., and Ren, D. (2014). The voltage-gated sodium channel TPC1 confers endolysosomal excitability. Nat. Chem. Biol. *10*, 463–469. https://doi.org/10.1038/nchembio.1522.

16. Johnson, D.E., Ostrowski, P., Jaumouillé, V., and Grinstein, S. (2016). The position of lysosomes within the cell determines their luminal pH. J. Cell Biol. *212*, 677–692. https://doi.org/10.1083/jcb.201507112.

17. Kornhuber, J., Tripal, P., Reichel, M., Mühle, C., Rhein, C., Muehlbacher, M., Groemer, T.W., and Gulbins, E. (2010). Functional Inhibitors of Acid Sphingomyelinase (FIASMAs): a novel pharmacological group of drugs with broad clinical applications. Cell. Physiol. Biochem. *26*, 9–20. https://doi.org/10.1159/000315101.

18. Trapp, S., Rosania, G.R., Horobin, R.W., and Kornhuber, J. (2008). Quantitative modeling of selective lysosomal targeting for drug design. Eur. Biophys. J. *37*, 1317–1328. https://doi.org/10.1007/s00249-008-0338-4.

19. Anand, A., Liu, B., Dicroce Giacobini, J., Maeda, K., Rohde, M., and Jäättelä, M. (2019). Cell death induced by cationic amphiphilic drugs depends on lysosomal Ca(2+) release and cyclic AMP. Mol. Cancer Therapeut. *18*, 1602–1614. https://doi.org/10.1158/1535-7163.MCT-18-1406.

20. Petersen, N.H.T., Olsen, O.D., Groth-Pedersen, L., Ellegaard, A.M., Bilgin, M., Redmer, S., Ostenfeld, M.S., Ulanet, D., Dovmark, T.H., Lønborg, A., et al. (2013). Transformation-associated changes in sphingolipid metabolism sensitize cells to lysosomal cell death induced by inhibitors of acid sphingomyelinase. Cancer Cell *24*, 379–393. https://doi.org/10.1016/j.ccr.2013.08.003.

21. Nielsen, I.Ø., Groth-Pedersen, L., Dicroce-Giacobini, J., Jonassen, A.S.H., Mortensen, M., Bilgin, M., Schmiegelow, K., Jäättelä, M., and Maeda, K. (2020). Cationic amphiphilic drugs induce elevation in lysoglycerophospholipid levels and cell death in leukemia cells. Metabolomics *16*, 91. https://doi.org/10.1007/s11306-020-01710-1.

22. Groth-Pedersen, L., Ostenfeld, M.S., Høyer-Hansen, M., Nylandsted, J., and Jäättelä, M. (2007). Vincristine induces dramatic lysosomal changes and sensitizes cancer cells to lysosome-destabilizing siramesine. Cancer Res. *67*, 2217–2225. https://doi.org/10.1158/0008-5472.CAN-06-3520.

23. Ostenfeld, M.S., Høyer-Hansen, M., Bastholm, L., Fehrenbacher, N., Olsen, O.D., Groth-Pedersen, L., Puustinen, P., Kirkegaard-Sørensen, T., Nylandsted, J., Farkas, T., and Jäättelä, M. (2008). Anti-cancer agent siramesine is a lysosomotropic detergent that induces cytoprotective autophagosome accumulation. Autophagy *4*, 487–499. https://doi.org/10.4161/auto.5774.

24. Sukhai, M.A., Prabha, S., Hurren, R., Rutledge, A.C., Lee, A.Y., Sriskanthadevan, S., Sun, H., Wang, X., Skrtic, M., Seneviratne, A., et al. (2013). Lysosomal disruption preferentially targets acute myeloid leukemia cells and progenitors. J. Clin. Invest. *123*, 315–328. https://doi.org/10.1172/JCI64180.

25. Jahchan, N.S., Dudley, J.T., Mazur, P.K., Flores, N., Yang, D., Palmerton, A., Zmoos, A.F., Vaka, D., Tran, K.Q.T., Zhou, M., et al. (2013). A drug repositioning approach identifies tricyclic antidepressants as inhibitors of small cell lung cancer and other neuroendocrine tumors. Cancer Discov. *3*, 1364–1377. https://doi.org/10.1158/2159-8290.CD-13-0183.

26. Ranjan, A., Gupta, P., and Srivastava, S.K. (2016). Penfluridol: an antipsychotic agent suppresses metastatic tumor growth in triple-negative breast cancer by inhibiting integrin signaling Axis. Cancer Res. *76*, 877–890. https://doi.org/10.1158/0008-5472.CAN-15-1233.

27. Shchors, K., Massaras, A., and Hanahan, D. (2015). Dual targeting of the autophagic regulatory circuitry in gliomas with repurposed drugs elicits cell-lethal autophagy and therapeutic benefit. Cancer Cell *28*, 456–471. https://doi.org/10.1016/j.ccell.2015.08.012.

28. Ellegaard, A.M., Dehlendorff, C., Vind, A.C., Anand, A., Cederkvist, L., Petersen, N.H.T., Nylandsted, J., Stenvang, J., Mellemgaard, A., Østerlind, K., et al. (2016). Repurposing cationic amphiphilic antihistamines for cancer treatment. EBioMedicine *9*, 130–139. https://doi.org/10.1016/j.ebiom.2016.06.013.

29. Verdoodt, F., Dehlendorff, C., Jäättelä, M., Strauss, R., Pottegård, A., Hallas, J., Friis, S., and Kjaer, S.K. (2020). Antihistamines and ovarian cancer survival: nationwide cohort study and in vitro cell viability assay. J. Natl. Cancer Inst. *112*, 964–967. https://doi.org/10.1093/jnci/djz217.

30. Fritz, I., Wagner, P., Broberg, P., Einefors, R., and Olsson, H. (2020). Desloratadine and loratadine stand out among common H1-antihistamines for association with improved breast cancer survival. Acta Oncol. *59*, 1103–1109. https://doi.org/10.1080/0284186X.2020.1769185.

31. Fritz, I., Wagner, P., and Olsson, H. (2021). Improved survival in several cancers with use of H1-antihistamines desloratadine and loratadine. Transl. Oncol. *14*, 101029. https://doi.org/10.1016/j.tranon.2021.101029.

32. Ermakova, Y.G., Pak, V.V., Bogdanova, Y.A., Kotlobay, A.A., Yampolsky, I.V., Shokhina, A.G., Panova, A.S., Marygin, R.A., Staroverov, D.B., Bilan, D.S., et al. (2018). SypHer3s: a genetically encoded fluorescent ratiometric probe with enhanced brightness and an improved dynamic range. Chem. Commun. *54*, 2898–2901. https://doi.org/10.1039/c7cc08740c.

33. Chen, T.W., Wardill, T.J., Sun, Y., Pulver, S.R., Renninger, S.L., Baohan, A., Schreiter, E.R., Kerr, R.A., Orger, M.B., Jayaraman, V., et al. (2013). Ultrasensitive fluorescent proteins for imaging neuronal activity. Nature *499*, 295–300. https://doi.org/10.1038/nature12354.

34. Odaka, H., Arai, S., Inoue, T., and Kitaguchi, T. (2014). Genetically-encoded yellow fluorescent cAMP indicator with an expanded dynamic range for dual-color imaging. PLoS One *9*, e100252. https://doi.org/10.1371/journal.pone.0100252.

35. Aits, S., Kricker, J., Liu, B., Ellegaard, A.M., Hämälistö, S., Tvingsholm, S., Corcelle-Termeau, E., Høgh, S., Farkas, T., Holm Jonassen, A., et al. (2015). Sensitive detection of lysosomal membrane permeabilization by lysosomal galectin puncta assay. Autophagy *11*, 1408–1424. https://doi.org/10.1080/15548627.2015.1063871.

36. Barber, D.L., and Ganz, M.B. (1992). Guanine nucleotides regulate beta-adrenergic activation of Na-H exchange independently of receptor coupling to Gs. J. Biol. Chem. *267*, 20607–20612.

37. Fonseca, B.D., Diering, G.H., Bidinosti, M.A., Dalal, K., Alain, T., Balgi, A.D., Forestieri, R., Nodwell, M., Rajadurai, C.V., Gunaratnam, C., et al. (2012). Structure-activity analysis of niclosamide reveals potential role

for cytoplasmic pH in control of mammalian target of rapamycin complex 1 (mTORC1) signaling. J. Biol. Chem. *287*, 17530–17545. https://doi.org/10.1074/jbc.M112.359638.

38. Jurgeit, A., McDowell, R., Moese, S., Meldrum, E., Schwendener, R., and Greber, U.F. (2012). Niclosamide is a proton carrier and targets acidic endosomes with broad antiviral effects. PLoS Pathog. *8*, e1002976. https://doi.org/10.1371/journal.ppat.1002976.
39. Lorkowski, S.W., Brubaker, G., Gulshan, K., and Smith, J.D. (2018). V-ATPase (vacuolar ATPase) activity required for ABCA1 (ATP-Binding cassette protein A1)-mediated cholesterol efflux. Arterioscler. Thromb. Vasc. Biol. *38*, 2615–2625. https://doi.org/10.1161/ATVBAHA.118.311814.
40. Samsonov, A., Zenser, N., Zhang, F., Zhang, H., Fetter, J., and Malkov, D. (2013). Tagging of genomic STAT3 and STAT1 with fluorescent proteins and insertion of a luciferase reporter in the cyclin D1 gene provides a modified A549 cell line to screen for selective STAT3 inhibitors. PLoS One *8*, e68391. https://doi.org/10.1371/journal.pone.0068391.
41. Chen, C.L., Hsieh, F.C., Lieblein, J.C., Brown, J., Chan, C., Wallace, J.A., Cheng, G., Hall, B.M., and Lin, J. (2007). Stat3 activation in human endometrial and cervical cancers. Br. J. Cancer *96*, 591–599. https://doi.org/10.1038/sj.bjc.6603597.
42. Schütz, A., Röser, K., Klitzsch, J., Lieder, F., Aberger, F., Gruber, W., Mueller, K.M., Pupyshev, A., Moriggl, R., and Friedrich, K. (2015). Lung adenocarcinomas and lung cancer cell lines show association of MMP-1 expression with STAT3 activation. Transl. Oncol. *8*, 97–105. https://doi.org/10.1016/j.tranon.2015.02.002.
43. Bai, E., Yang, L., Xiang, Y., Hu, W., Li, C., Lin, J., Dai, X., Liang, G., Jin, R., and Zhao, C. (2018). L61H46 shows potent efficacy against human pancreatic cancer through inhibiting STAT3 pathway. Cancer Manag. Res. *10*, 565–581. https://doi.org/10.2147/CMAR.S159090.
44. Gough, D.J., Corlett, A., Schlessinger, K., Wegrzyn, J., Larner, A.C., and Levy, D.E. (2009). Mitochondrial STAT3 supports Ras-dependent oncogenic transformation. Science *324*, 1713–1716. https://doi.org/10.1126/science.1171721.
45. Wegrzyn, J., Potla, R., Chwae, Y.J., Sepuri, N.B.V., Zhang, Q., Koeck, T., Derecka, M., Szczepanek, K., Szelag, M., Gornicka, A., et al. (2009). Function of mitochondrial Stat3 in cellular respiration. Science *323*, 793–797. https://doi.org/10.1126/science.1164551.
46. Ferrajoli, A., Faderl, S., Van, Q., Koch, P., Harris, D., Liu, Z., Hazan-Halevy, I., Wang, Y., Kantarjian, H.M., Priebe, W., and Estrov, Z. (2007). WP1066 disrupts Janus kinase-2 and induces caspase-dependent apoptosis in acute myelogenous leukemia cells. Cancer Res. *67*, 11291–11299. https://doi.org/10.1158/0008-5472.CAN-07-0593.
47. Song, H., Wang, R., Wang, S., and Lin, J. (2005). A low-molecular-weight compound discovered through virtual database screening inhibits Stat3 function in breast cancer cells. Proc. Natl. Acad. Sci. USA *102*, 4700–4705. https://doi.org/10.1073/pnas.0409894102.
48. Schust, J., Sperl, B., Hollis, A., Mayer, T.U., and Berg, T. (2006). Stattic: a small-molecule inhibitor of STAT3 activation and dimerization. Chem. Biol. *13*, 1235–1242. https://doi.org/10.1016/j.chembiol.2006.09.018.
49. Rouillard, A.D., Gundersen, G.W., Fernandez, N.F., Wang, Z., Monteiro, C.D., McDermott, M.G., and Ma'ayan, A. (2016). The harmonizome: a collection of processed datasets gathered to serve and mine knowledge about genes and proteins. Database *2016*, baw100. https://doi.org/10.1093/database/baw100.
50. Demidenko, E., and Miller, T.W. (2019). Statistical determination of synergy based on Bliss definition of drugs independence. PLoS One *14*, e0224137. https://doi.org/10.1371/journal.pone.0224137.
51. Foghsgaard, L., Wissing, D., Mauch, D., Lademann, U., Bastholm, L., Boes, M., Elling, F., Leist, M., and Jäättelä, M. (2001). Cathepsin B acts as a dominant execution protease in tumor cell apoptosis induced by tumor necrosis factor. J. Cell Biol. *153*, 999–1010. https://doi.org/10.1083/jcb.153.5.999.
52. Degterev, A., Hitomi, J., Germscheid, M., Ch'en, I.L., Korkina, O., Teng, X., Abbott, D., Cuny, G.D., Yuan, C., Wagner, G., et al. (2008). Identification of RIP1 kinase as a specific cellular target of necrostatins. Nat. Chem. Biol. *4*, 313–321. https://doi.org/10.1038/nchembio.83.
53. Skouta, R., Dixon, S.J., Wang, J., Dunn, D.E., Orman, M., Shimada, K., Rosenberg, P.A., Lo, D.C., Weinberg, J.M., Linkermann, A., and Stockwell, B.R. (2014). Ferrostatins inhibit oxidative lipid damage and cell death in diverse disease models. J. Am. Chem. Soc. *136*, 4551–4556. https://doi.org/10.1021/ja411006a.
54. Rafn, B., Nielsen, C.F., Andersen, S.H., Szyniarowski, P., Corcelle-Termeau, E., Valo, E., Fehrenbacher, N., Olsen, C.J., Daugaard, M., Egebjerg, C., et al. (2012). ErbB2-driven breast cancer cell invasion depends on a complex signaling network activating myeloid zinc finger-1-dependent cathepsin B expression. Mol. Cell *45*, 764–776. https://doi.org/10.1016/j.molcel.2012.01.029.
55. Olson, O.C., and Joyce, J.A. (2015). Cysteine cathepsin proteases: regulators of cancer progression and therapeutic response. Nat. Rev. Cancer *15*, 712–729. https://doi.org/10.1038/nrc4027.
56. Huynh, J., Chand, A., Gough, D., and Ernst, M. (2019). Therapeutically exploiting STAT3 activity in cancer - using tissue repair as a road map. Nat. Rev. Cancer *19*, 82–96. https://doi.org/10.1038/s41568-018-0090-8.
57. Yu, H., Lee, H., Herrmann, A., Buettner, R., and Jove, R. (2014). Revisiting STAT3 signalling in cancer: new and unexpected biological functions. Nat. Rev. Cancer *14*, 736–746. https://doi.org/10.1038/nrc3818.
58. Hutchins, A.P., Diez, D., and Miranda-Saavedra, D. (2013). Genomic and computational approaches to dissect the mechanisms of STAT3's universal and cell type-specific functions. JAK-STAT *2*, e25097. https://doi.org/10.4161/jkst.25097.
59. Carpenter, R.L., and Lo, H.W. (2014). STAT3 target genes relevant to human cancers. Cancers *6*, 897–925. https://doi.org/10.3390/cancers6020897.
60. Senichkin, V.V., Streletskaia, A.Y., Gorbunova, A.S., Zhivotovsky, B., and Kopeina, G.S. (2020). Saga of Mcl-1: regulation from transcription to degradation. Cell Death Differ. *27*, 405–419. https://doi.org/10.1038/s41418-019-0486-3.
61. Matsuyama, S., Llopis, J., Deveraux, Q.L., Tsien, R.Y., and Reed, J.C. (2000). Changes in intramitochondrial and cytosolic pH: early events that modulate caspase activation during apoptosis. Nat. Cell Biol. *2*, 318–325. https://doi.org/10.1038/35014006.
62. Jäättelä, M., Benedict, M., Tewari, M., Shayman, J.A., and Dixit, V.M. (1995). Bcl-x and Bcl-2 inhibit TNF and Fas-induced apoptosis and activation of phospholipase A2 in breast carcinoma cells. Oncogene *10*, 2297–2305.
63. Schneider, C.A., Rasband, W.S., and Eliceiri, K.W. (2012). NIH Image to ImageJ: 25 years of image analysis. Nat. Methods *9*, 671–675. https://doi.org/10.1038/nmeth.2089.

STAR★METHODS

KEY RESOURCES TABLE

REAGENT or RESOURCE	SOURCE	IDENTIFIER
Antibodies		
Rabbit anti-P-STAT3(Y705)	Cell Signaling Technology	Cat# 9145, RRID:AB_2491009
Rabbit anti-STAT3	Cell Signaling Technology	Cat# 4904, RRID:AB_331269
HRP-anti-α-Tubulin	Abcam	Cat# ab40742, RRID:AB_880625
Rabbit anti-CCND1	Millipore	Cat# 06–137, RRID:AB_2070695
Rabbit anti-P-STAT3(S727)	Abcam	Cat# ab86430, RRID:AB_10713219
Rabbit anti-P-STAT3(Y705)	Cell Signaling Technology	Cat# 9145, RRID:AB_2491009
Rabbit anti-Galectin 1	Abcam	Cat# ab25138, RRID:AB_2136615
Rabbit anti-BAX	Cell Signaling Technology	Cat# 2772, RRID:AB_10695870
Donkey anti-rabbit IgG, Alexa Fluor 594	Thermo Fisher Scientific	Cat# A-21207, RRID:AB_141637
Donkey anti-rabbit IgG, Alexa Fluor 488	Thermo Fisher Scientific	Cat# A-21206, RRID:AB_2535792
Anti-rabbit IgG from goat	Vector Laboratories	Cat# PI-1000, RRID:AB_2336198
Chemicals, peptides, and recombinant proteins		
Terfenadine	Sigma Aldrich	Cat# T9652
Niclosamide	Sigma Aldrich	Cat# N3510
Forskolin	Sigma Aldrich	Cat# 93049
Penfluridol	Sigma Aldrich	Cat# P3371
Astemizole	Sigma Aldrich	Cat# A2861
EIPA	Sigma Aldrich	Cat# A3085
Ebastine	Cayman Chemical	Cat# T9652
WP1066	Merk	Cat# N3510
STA-21	Selleckchem	Cat# S7951
Stattic	Abcam	Cat# Ab120952
Hoechst 33,342	Sigma Aldrich	Cat# B2261
Propidium iodide solution	Sigma Aldrich	Cat# P4864
Dextran, Fluorescein, 70,000 MW, Anionic	Thermo Fisher Scientific	Cat# D-1823
Dextran, Tetramethylrhodamine, 70,000 MW	Thermo Fisher Scientific	Cat# D-1818
BCECF-AM	Thermo Fisher Scientific	Cat# B1170
cOmplete Protease inhibitor Cocktail	Merk	Cat# 11697498001
Critical commercial assays		
TurboFectin 8.0	Origene	Cat# TF81001
Lipofectamine 3000	Thermo Fisher Scientific	Cat# L3000008
Lipofectamine RNAmax	Thermo Fisher Scientific	Cat# 13778075
NE-PER^{TM} Nuclear Extraction Kit	Thermo Fisher Scientific	Cat# 78833
Quant-X^{TM} One-Step qRT-PCR SYBR® Kit	Takara Bio	Cat# 638317
Pierce Gaussia Luciferase Glow Assay Kit	Thermo Fisher Scientific	Cat# 16160
Fluo 4, AM	Thermo Fisher Scientific	Cat# F-14201
Venor®GeM Classic PCR kit	MB Minerva biolabs	Cat# 11-1025G

(*Continued on next page*)

Continued		
REAGENT or RESOURCE	SOURCE	IDENTIFIER
Experimental models: Cell lines		
HeLa cervix carcinoma cell	ECACC	Cat# 93021013, RRID:CVCL_0030
HeLa NC4, STAT3 KO1, STAT3 KO11	Liu et al.[12]	N/A
PANC-1 pancreatic tumor cell	Creative Bioarray	CSC-C8212L
A549 non-small cell lung carcinoma cell	ATCC	Cat# CCL-185, RRID:CVCL_0023
A549 triple cell	Dmitry Malkov lab	N/A
MCF7 breast cancer cell, TNF-sensitive subclone S1	Jaattela et al.,[62]	N/A
Experimental models: Organisms/strains		
BALB/c nude mouse	Beijing Vital River Laboratory Animal Technology Co., Ltd	N/A
Oligonucleotides		
Primers for qPCR: See Table S1	This paper	N/A
Primers for cloning: See Table S1	This paper	N/A
siRNA targeting STAT3, P2RX4, ADCY1, TMEM165: See Table S1	This paper	N/A
Recombinant DNA		
pC1SypHer3S	Addgene	Cat# 108118
Flamindo2	Addgene	Cat# 73938
pLentiCMVIE-IRES-BlastR	Addgene	Cat# 119863
Software and algorithms		
Fiji	Schneider et al.[63]	ImageJ.net/Fiji
GraphPad Prism 8.0	GraphPad Prism	https://www.graphpad.com/scientific-software/prism/
Image Studio lite	Li-COR	https://www.licor.com/bio/image-studio-lite/
MetaXpress software	Molecular Device	https://www.moleculardevices.com/products/cellular-imaging-systems/acquisition-and-analysis-software/metaxpress#gref
Other		
Microlite^{TM1} Luminescence Microtiter 96-well plate	VWR Scientific	Cat# 62403-124
pHrodo Green-AM	Thermo Fisher Scientific	Cat# P35373
Live Cell Imaging Solution	Thermo Fisher Scientific	Cat# A14291DJ

RESOURCE AVAILABILITY

Lead contact

Dr. Marja Jaattela (mj@cancer.dk).

Materials availability

All cell lines, plasmids and other reagents generated in this study are available from the lead contact with a completed Materials Transfer Agreement if there is potential for commercial application.

Data and code availability

- The RNA-seq data has been deposited to the public data depository CNGB Sequence Archive (https://db.cngb.org/cnsa/) : CNP0003232.
- This paper does not report original code.
- Other data reported in this paper is available from the lead contact upon request.

EXPERIMENTAL AND SUBJECT DETAILS

Cell lines

Human A549 non-small cell lung carcinoma (male) were obtained from American Type Culture Collection (ATCC). HeLa cervix carcinoma (female) cells were obtained from ECACC. PANC-1 pancreatic tumor (male) cells were purchased from Creative Bioarray. A549-triple cells, where red fluorescent protein (RFP) has been knocked in the NH_2-terminus of the endogenous STAT3 and secreted luciferase has been knocked in after endogenous CCND1 promoter,[40] were kindly provided by Dmitry Malkov (Sigma-Aldrich, St. Louis, Missouri, USA). HeLa STAT3 NC4, KO1, and KO11 were obtained by CRISPR previously.[12] The TND-sensitive S1 subclone of MCF7 cells has been described previously.[62] All cell lines were regularly tested and found negative for mycoplasma using VenorGeM Classic PCR kit. HeLa cells were cultured in DMEM supplemented with 10% heat-inactivated fetal calf serum and penicillin/streptomycin. A549 and PANC-1 cells were cultured in DMEM supplemented with 2mM Glutamine, 10% heat-inactivated fetal calf serum and penicillin/streptomycin.

Animals

Six weeks old nude female BALB/c mice were purchased from Beijing Vital River Laboratory Animal Technology Co., Ltd. Mice were maintained in a temperature (~23°C) and humidity (~50%) controlled room, with free access to the standard diet and water, with a 12/12-h light-dark cycle. All of the procedures were approved by the Committee of Experimental Animals of the Ocean University of China and conformed to the NIH Guide for the Care and Use of Laboratory Animals.

METHOD DETAILS

Transfections

If not otherwise stated, plasmid transfections were performed by using TurboFectin 8.0 (Origene, CAT#: TF81001) or Lipofectamine 3000 transfection agents (Thermo Fisher Scientific, Cat# L3000008) according to the manufacturer's instructions. siRNA transfections were performed by using Lipofectamine RNAmax (Thermo Fisher Scientific, Cat# 13778075) according to the manufacturer's instructions.

siRNA

siRNA targeting STAT3 is GAAUCACGCCUUCUACAGA/UCUGUAGAAGGCGUGAUUC. siRNAs targeting P2X4 are CAAGUCGUG CAUUUAUGAUtt/AUCAUAAAUGCACGACUUGtt, GUCCUCUACUGCAUGAAGAtt/UCUUCAUGCAGUAGAGGACtt. siRNA targeting ADCY1 was purchased from Sigma Aldrich (Identifier: SASI_Hs01_00226331). siRNAs targeting TMEM165 are GUAUCUG AAUUG GGUGAUAtt/UAUCACCCAAUUCAGAUACaa, CAGGGUCUAUACAUACUAUtt/AUAGUAUGUAUAGACCCUGaa, GCAUA ACAGUACCUCAGAAtt/UUCUGAGGUACUGUUAUGCtt.

Western blot

Cells were lysed in Laemmli sample buffer (125 mM Tris, pH 6.7, 20% glycerol, 140mM SDS) supplemented with complete protease inhibitor cocktail (Merk, Cat# 11697498001). After addition of 0.05M dithiothreitol and bromophenol blue, boiling and separation by 4%–20% gradient SDS-polyacrylamide gel electrophoresis, proteins were transferred onto polyvinylidene difluoride membranes using Bio-Rad Trans-Blot Turbo system. Membranes were blocked with PBS containing 5% milk and 0.1% Tween 20, and stained with the indicated primary antibodies and appropriate peroxidase-conjugated secondary antibody. The signal was detected with Clarity Western ECL Substrate and Luminescent Image Reader, and quantified by densitometry with Image Studio Lite software.

Immunostaining

Cells grown on coverslips and fixed in 4% paraformaldehyde in DPBS for 20 min were permeabilized with 0.1% saponin in DPBS for 10 min and blocked in 5% goat serum in DPBS for 10 min before staining with indicated primary antibodies followed by appropriate Alexa Fluor488-or Alexa Fluor594-coupled secondary antibodies. Nuclei were labeled either with 5 mg/ml Hoechst 33,342 or DAPI in the Prolong Gold antifade mounting medium.

Imaging

Live-cell images and immunostaining images were taken by confocal microscope LSM700 with Plan-Apochromat 63×/1.40 Oil DIC M27 objective and Zen 2010 software (all equipment and software from Carl Zeiss, Jena, Germany). Pinholes were set so that the section thickness was equal for all channels and ≤1 AU. Cell contours (n > 20) were defined manually and green and red thresholds were set up in a single-channel mode and retained for all samples in an experiment. All the images were analyzed by ImageJ (Fiji) software (ImageJ.net/Fiji). Time series of high-throughput images were taken by ImageXpress Micro System with temperature and environmental control. 40x magnification was applied. The image analysis was performed with MetaXpress software.

Real-time PCR

Real-time PCR was performed by using Quant-X One-Step qRT-PCR SYBR Kit (Takara, Cat. 638317) according to the manufacturer's instructions. Primers' information is shown in Table S1.

Fluo 4 staining

To measure relative levels of cytosolic free Ca^{2+}, cells were stained with 3 mmol/L Fluo-4-AM for 25 min, washed twice with Dulbecco phosphate-buffered saline (DPBS), resuspended in DPBS without Ca^{2+}, Mg^{2+} (if not otherwise indicated) plus 20 mmol/L HEPES and maintained at 37C while treating the cells as indicated and analyzing by BD FACSVerse flow cytometer (FL-1 channel).

Luciferase assay

Luciferase assay was performed by using. In brief, A549 triple cells were plated in a 96-well plate with 10^4 cells per well. 24 h later, change medium to terfenadine or ebastine-containing medium and treat cells for 1, 2, and 4 h respectively. Collect the medium containing secreted luciferase driven by CCND1's promoter. Following the manufacturer's instruction of Pierce Gaussia Luciferase Glow Assay Kit (Cat. 16160), set up reactions in Microlite^{TM1} Luminescence Microtiter 96-well plate (VWR Scientific Products, Cat. 62,403-124) and recorded the luminance by Spectromax ID3 plate reader (Molecular Devices). Meanwhile, after nuclear staining of cells by Hoechst 33,342 (Sigma, Cat. B2261), the number of cells in each well was counted by Celigo Image Cytometer (Nexcelom Bioscience). Luminance normalized by cell number was displayed as a readout of STAT3's transcriptional activity.

Lysosomal pH measurement

To estimate the relative lysosomal pH, subconfluent cells were loaded with 2.5 mg/mL pH-sensitive FITC coupled to 70 kDa dextran and 2.5 mg/mL pH-insensitive tetramethylrhodamine (TMR) coupled to 70 kDa dextran for 18 h, washed, and chased in fresh medium for 5 h. The medium was changed to Live Cell Imaging Solution (Thermo Fisher Scientific, Cat. A14291DJ) before image acquisition by LSM700 Confocal Laser Scanning Microscope (Zeiss). Images were analyzed using ImageJ software.

Cytosolic pH measurement

Plate HeLa SypHer3S stable transfected cells into 96-well plate. Then treat the cells with different drugs for a certain time the next day. Change the medium to Live Cell Imaging Solution (Thermo Fisher Scientific, Cat. A14291DJ) and take images of the cells in ImageXpress high-content platform. Standard curves used to estimate cytosolic pH were created by a similar analysis of cells incubated with a series of pH calibration buffers (pH 4.5, 5.5, 6.5, and 7.5) supplemented with 10 μM valinomycin and 10 μM nigericin (Intracellular pH Calibration Kit) for 5 min. Alternatively, cells washed with Live Cell Imaging Solution were incubated for 30 min in 37 °C in the same solution containing 1:1,000 dilution of pHrodo Green AM Intracellular pH Indicator (Thermo Fisher Scientific, Cat. P35373) and 1:100 dilution of PowerLoad concentrate, washed with Live Cell Imaging Solution, and analyzed by ImageXpress high-content platform.

To confirm the pH determination with ratio-metric quantification, 5 μM BCECF-AM (Thermo Fisher Scientific, Cat. B1170) in DMEM was added to cells for 30 min. Then replace the medium with Ringer solution (115 mM NaCl, 5 mM KCl, 1 mM Na_2HPO_4, 1 mM $CaCl_2$, 0.5 mM $MgCl_2$, pH = 7.4) and take images of the cells with ex/em of both 470/520 and 445/520 in the ImageXpress high-content platform. Standard curves used to estimate cytosolic pH were created by a similar analysis of cells incubated with a series of pH calibration buffers (140 mM KCl, 1 mM K_2HPO_4, 1 mM $CaCl_2$, 0.5 mM $MgCl_{2,}$ pH 6, 6.5, 7, and 7.5) supplemented with 10 μM valinomycin and 10 μM nigericin for 5 min.

Ammonium prepulse assay

Stain the cells with 5 μM BCECF-AM as above. Then wash the cells once with HCO_3^- Ringer solution (115 mM NaCl. 5 mM KCl, 1 mM Na_2HPO_4, 1 mM $CaCl_2$, 24 mM $NaHCO_3$). Take images of cells with ex/em of 470/520 and 445/520 in the ImageXpress high-content platform every 20 s for 5 min in total. Thereafter, replace the Ringer solution with 20 mM NH_4Cl Ringer solution and take images of cells again every 20 s for 5 min in total. Lastly, NH_4Cl Ringer solution is replaced with HCO_3^- Ringer solutions containing DMSO, 15 μM Ebastine or 25 μM EIPA respectively. Immediately after the last change of solution, take images of cells every 20 s for 5 min in total. The obtained fluorescence readout is converted into pH values according to the standard curve and the real-time pH monitoring curve is plotted.

Nuclear fractionation

Nuclear extraction was performed according to the manufactural instruction of NE-PER Nuclear Extraction Kit (Cat# 78833).

Cell death

5000 cells were plated in each well of a 96-well plate. Subsequently, cells were treated as indicated and cell death was measured after 10 min incubation with 0.2 μg/mL propidium iodide (Sigma, Cat# P4864) and 2.5 μg/mL Hoechst (Sigma, Cat# B2261) at 37°C using Celigo Imaging Cytometer (Nexcelom Bioscience) according to the manufacture's instructions.

Synergy score determination

For each combination of two drugs, synergy scores were estimated using the Bliss independence model.[50] We used the model-free statistical determination of synergy approach described in [Demidenko] for dose-response relationships.

RNA seq

The mRNA and non-coding RNAs were enriched by removing rRNA with RNaseH. Target RNAs were fragmented into short fragments in the fragmentation buffer, and cDNAs were synthesized using the RNA fragments as templates for N6 random primer, followed by end reparation and ligation to adapters. The quantity and quality of the cDNA libraries were assessed using an Agilent 2100 BioAnalyzer (Agilent Technologies). Finally, the libraries were sequenced on the BGISEQ-500 with 50 single-end reads. Sequencing reads that contained adapters had low quality or aligned to rRNA were filtered off before mapping. Clean reads were aligned to the hg19 UCSC RefSeq (RNA sequences, GRCh37) using bowtie2. FPKM values were obtained by transforming mapped transcript reads using RSEM. Differential expression analysis was performed by DESeq2. Differentially expressed genes (DEGs) were defined as genes with fold change ≥ 1.5 and p value ≤ 0.05. Clean reads were mapped to the hg19 genome using hisat2.

Xenografts

Female 6-weeks old BALB/c nude mice were subcutaneously injected with $5x10^6$ A549 cells. Eight days post tumor implantation, mice were randomly divided into 4 groups and intraperitoneally treated with vehicle, ebasine (30 mg/kg), WP1066 (20 mg/kg), or ebastine (30 mg/kg) and WP1066 (20 mg/kg), respectively. Both ebasine and WP1066 were dissolved in DMSO: PEG300 at the volume ratio of 2:8, and given to mice 3 times a week. Bodyweight and tumor volume was recorded at the days of drug administration. The tumor weight and spleen index were recorded at the end of the experiments.

QUANTIFICATION AND STATISTICAL ANALYSIS

Graphs were generated by GraphPad Prism 8.0. All bar figures are presented as average ±SD. One-way ANOVA analyses with Dunnet multiple comparisons were performed for single grouped data while two-way ANOVA analyses with Turkey multiple comparisons were performed for multi-grouped data. Experiments were performed with at least three biologically independent replicates unless stated otherwise. Statistical significance was defined as a p value equal to or less than 0.05. If displayed as symbols, p values are depicted as: *p $\leq$ 0.05, **p $\leq$ 0.01, ***p $\leq$ 0.001.

Cell Reports, Volume *42*

Supplemental information

Cationic amphiphilic antihistamines inhibit STAT3 via Ca^{2+}-dependent lysosomal H^+ efflux

Bin Liu, Ran Chen, Yidan Zhang, Jinrong Huang, Yonglun Luo, Susanne Rosthøj, Chenyang Zhao, and Marja Jäättelä

Supplementary Information

Figure S1. CADs induce rapid cytosolic acidification in cancer cells. Related to figure 1.

A. Loss of plasma membrane integrity of HeLa cells treated with 6 µM terfenadine or 15 µM ebastine for indicated times, stained with membrane-permeable Hoechst (all cells) and propidium iodide (cells with plasma membrane permeabilization) and analyzed by Celigo Imaging Cytometer.

B. Kinetics of SypHer3s FLIs in HeLa cells left untreated or treated with 6 µM terfenadine or 15 µM ebastine for indicated times and analyzed as in Figure 1A.

C. Representative images of Ebastine-treated HeLa cells presented in Figure 1A (*right* panel) with arrows pointing to cells with typical CAD-induced changes. Scale bars, 30 µM.

D. SypHer3s FLI in HeLa-SypHer3s cells left untreated or treated with 10 µM niclosamide (positive control for cytosolic acidification), 6 µM terfenadine, 15 µM ebastine, 10 µM astemizole or 6 µM penfluridol for 1 h prior to the flow cytometry analysis.

E. SypHer3s FLI in A549-SypHer3s cells treated for 2 h with DMSO, 10 µM niclosamide (positive control), 6 µM terfenadine, or 15 µM ebastine and analyzed by flow cytometry.

F. Quantification of LGALS1/galectin 1 puncta (leaky lysosomes) in A549 cells treated with 6 µM terfenadine or 15 µM ebastine for 0 - 8 h.

Error bars, SD of three triplicate independent experiments with ≥ 10000 (A, D, and E) or ≥10 (B and F) randomly chosen cells analyzed in each sample. *, $P < 0.05$; **, $P < 0.01$; ***, $P < 0.001$ as analyzed by one-way Anova with Tukey multiple comparison (D and E) or two-way Anova with Dunnet multiple comparison (A, B and F).

Figure S2. CAD-induced cytosolic acidification depends on P2RX4 but not cAMP generation. Related to figure 3.

A, B. Cytosolic pH values of MCF7-SypHer3s cells treated with indicated siRNAs for 72 and with DMSO or 6 µM terfenadine for the last 2 h (*left*) were analyzed as in Figure 1C. Representative (n=3) immunoblots of indicated proteins in siRNA-treated MCF7-SypHer3S cells (*right*).

C. Flamindo (*left*) and SypHer3s (*right*) FLIs in HeLa cells treated with indicated concentrations of drugs for 1 h (*left*) or 2 h (*right*) and analyzed by flow cytometry.

Error bars, SD of three triplicate independent experiments with ≥10 (A, B) or ≥ 10000 (C) randomly chosen cells analyzed in each sample. *, $P < 0.05$; **, $P < 0.01$; ***, $P < 0.001$ as analyzed by one-way Anova with Tukey multiple comparison (C) or two-way Anova with Dunnet multiple comparison (A, B).

Figure S3. CADs trigger lysosomal translocation of STAT3. Related to figure 4.

Representative images of A549 triple cells with RFP-tagged endogenous STAT3 transfected with LAMP1-BFP and treated with DMSO, 6 µM terfenadine, or 15 µM ebastine for 2 h. Scale bars, 10 µm. For quantification see Figure 4B.

Figure S4. CADs reduce Y705-STAT3 phosphorylation and CCND1 expression. Related to figure 5.

A. Quantification of P-S727-STAT3 / STAT3 and STAT3 / TUBA1 ratios in immunoblots of lysates of HeLa cells left untreated or treated with 6 µM terfenadine, 15 µM ebastine, 10 µM astemizole or 6 µM penfluridol for indicated times. See Figure 5A for representative blots.

B-E. Representative immunoblots of indicated proteins from A549 (B) and PANC1 (D) cells treated with CADs as indicated and the quantification of blots from three independent experiments (C and E).

F. Quantification of the amount of dephosphorylated STAT3 according to Fig 5F.

Error bars, SD of three independent experiments. *, $P < 0.05$; **, $P < 0.01$; ***, $P < 0.001$ as analyzed by by two-way Anova with Dunnet multiple comparison.

Figure S5. Examples of terfenadine- and ebastine-regulated STAT3 target genes. Related to figure 6.

A and B. Box plots for the expression of indicated STAT3 target genes in HeLa cells treated with 6 µM terfenadine (A) or 15 µM ebastine (B) for 0, 2, or 8 h. Boxes, median and interquartile range of the values from three independent RNASeq analyses. Whiskers, variability outside the quartiles. TPM, transcripts per million.

C and D. KEGG enrichment analysis of terfenadine and ebastine specifically-downregulated STAT3 target genes. Fisher's test p values are displayed.

Significance of statistics is indicated when fold change is bigger than 1.5 and adjusted P value meet criteria: *, $P < 0.05$; **, $P < 0.01$; ***, $P<0.001$. Data is based on three independent RNAseq experiments.

Figure S6. CAD-induced cytosolic acidification exerts a synergistic anti-tumor effect with WP1066. Related to figure 7.

A. Representative immunoblots of indicated proteins in HeLa cells with indicated treatments (*left*) and the quantification of blots from three independent experiments (*right*).

B. Luciferase activity in the media of A549 triple cells expressing CCND1 promoter-driven secreted luciferase. Cells were left untreated or treated with 4 µM terfenadine, or 8 µM ebastine for indicated times.

C. Cell death of HeLa cells with indicated treatments for 24 h determined by propidium iodide and Hoechst-33342 staining employing Celígo Imaging Cytometer.

D. Cell death of A549 cells with indicated treatments for 24 h determined by propidium iodide and Hoechst-33342 staining employing Celígo Imaging Cytometer. Synergy score was determined by the Bliss independent model (See methods).

E. SypHer3s FLI in HeLa cells treated by 4 µM terfenadine, 5 µM WP1066, or the combination of both drugs for 1 h and analyzed by FACS.

Error bars, SD of three independent triplicate experiments with ≥1000 cells analyzed in each condition.

*, $P < 0.05$; **, $P < 0.01$; ***, $P < 0.001$ as analyzed by one way Anova with Turkey multiple comparison (E) or two-way Anova with Dunnet multiple comparison (A-D).

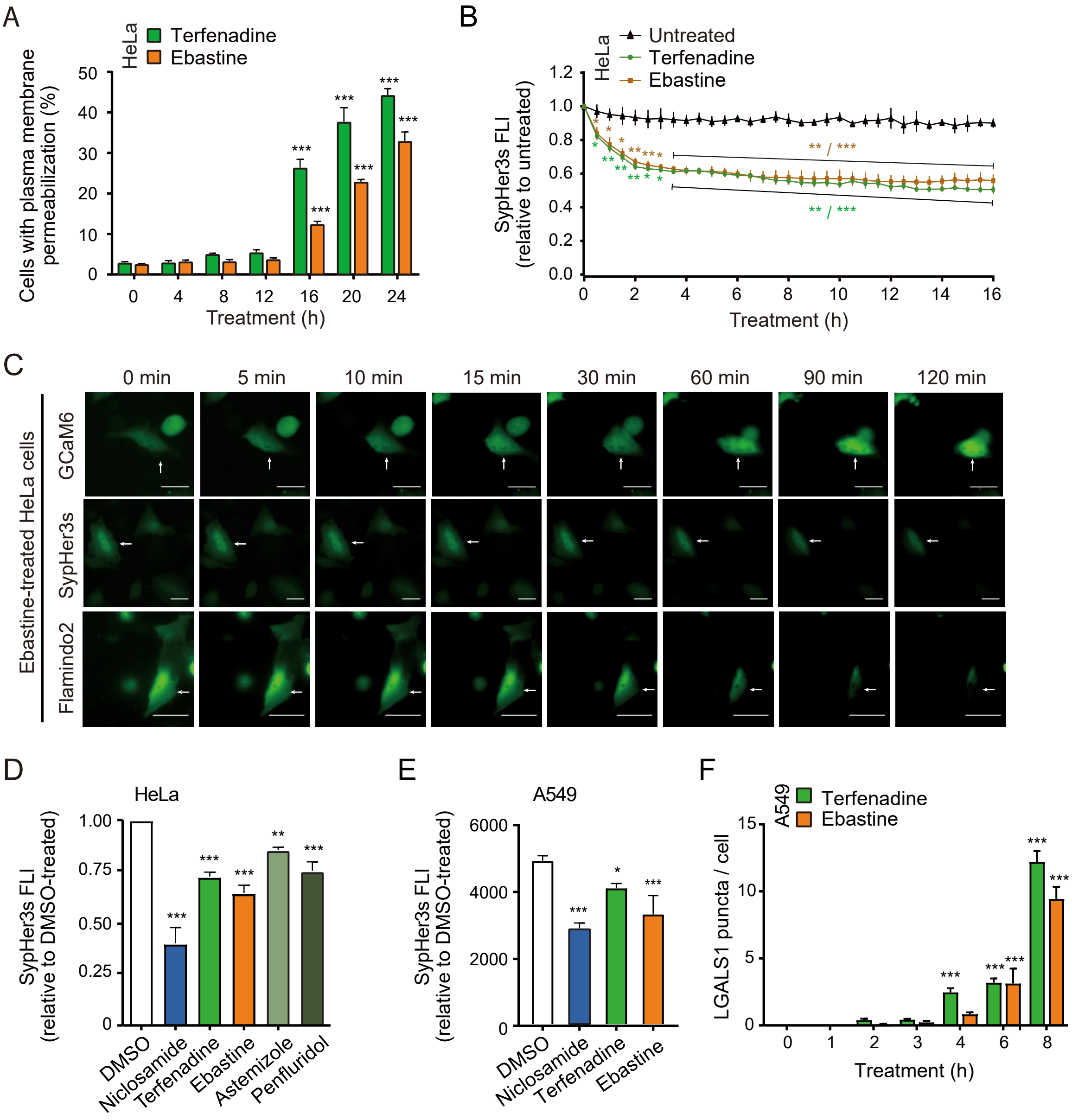
A
HeLa
Terfenadine
Ebastine
Cells with plasma membrane permeabilization (%)
Treatment (h)
B
HeLa
Untreated
Terfenadine
Ebastine
SypHer3s FLI (relative to untreated)
Treatment (h)
C
0 min
5 min
10 min
15 min
30 min
60 min
90 min
120 min
Ebastine-treated HeLa cells
GCaM6
SypHer3s
Flamindo2
D
HeLa
SypHer3s FLI (relative to DMSO-treated)
DMSO
Niclosamide
Terfenadine
Ebastine
Astemizole
Penfluridol
E
A549
SypHer3s FLI (relative to DMSO-treated)
DMSO
Niclosamide
Terfenadine
Ebastine
F
A549
Terfenadine
Ebastine
LGALS1 puncta / cell
Treatment (h)

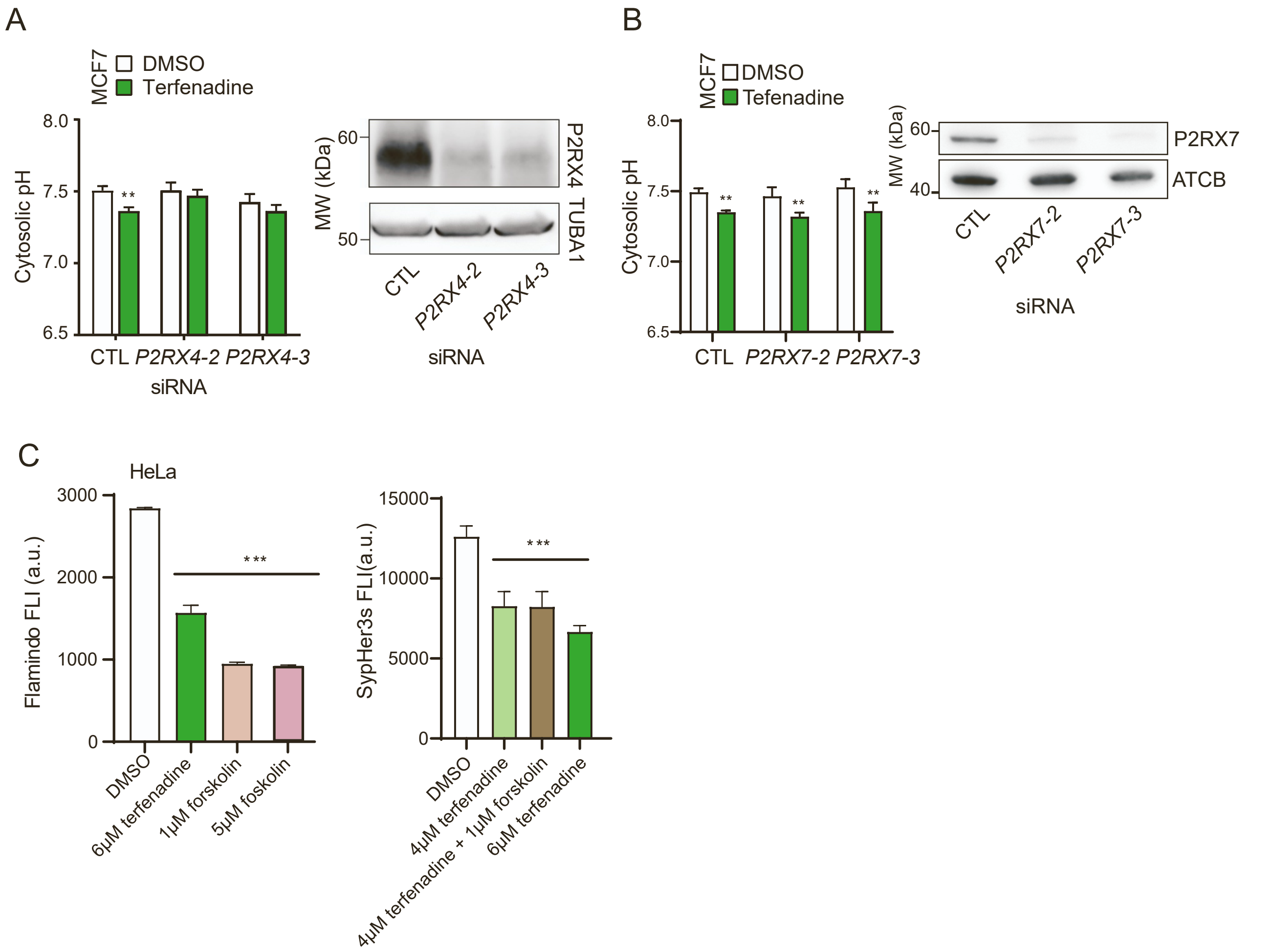
A
MCF7
DMSO
Terfenadine
Cytosolic pH
8.0
7.5
7.0
6.5
**
CTL P2RX4-2 P2RX4-3
siRNA
MW (kDa)
60
50
P2RX4
TUBA1
CTL
P2RX4-2
P2RX4-3
siRNA
B
MCF7
DMSO
Tefenadine
Cytosolic pH
8.0
7.5
7.0
6.5
**
**
**
CTL P2RX7-2 P2RX7-3
MW (kDa)
60
40
P2RX7
ATCB
CTL
P2RX7-2
P2RX7-3
siRNA
C
HeLa
Flamindo FLI (a.u.)
3000
2000
1000
0
***
DMSO
6μM terfenadine
1μM forskolin
5μM foskolin
SypHer3s FLI(a.u.)
15000
10000
5000
0
***
DMSO
4μM terfenadine
4μM terfenadine + 1μM forskolin
6μM terfenadine

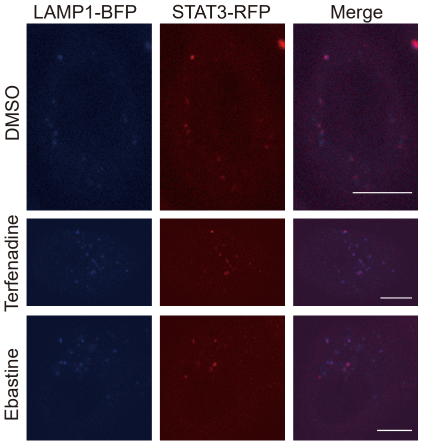
LAMP1-BFP
STAT3-RFP
Merge
DMSO
Terfenadine
Ebastine

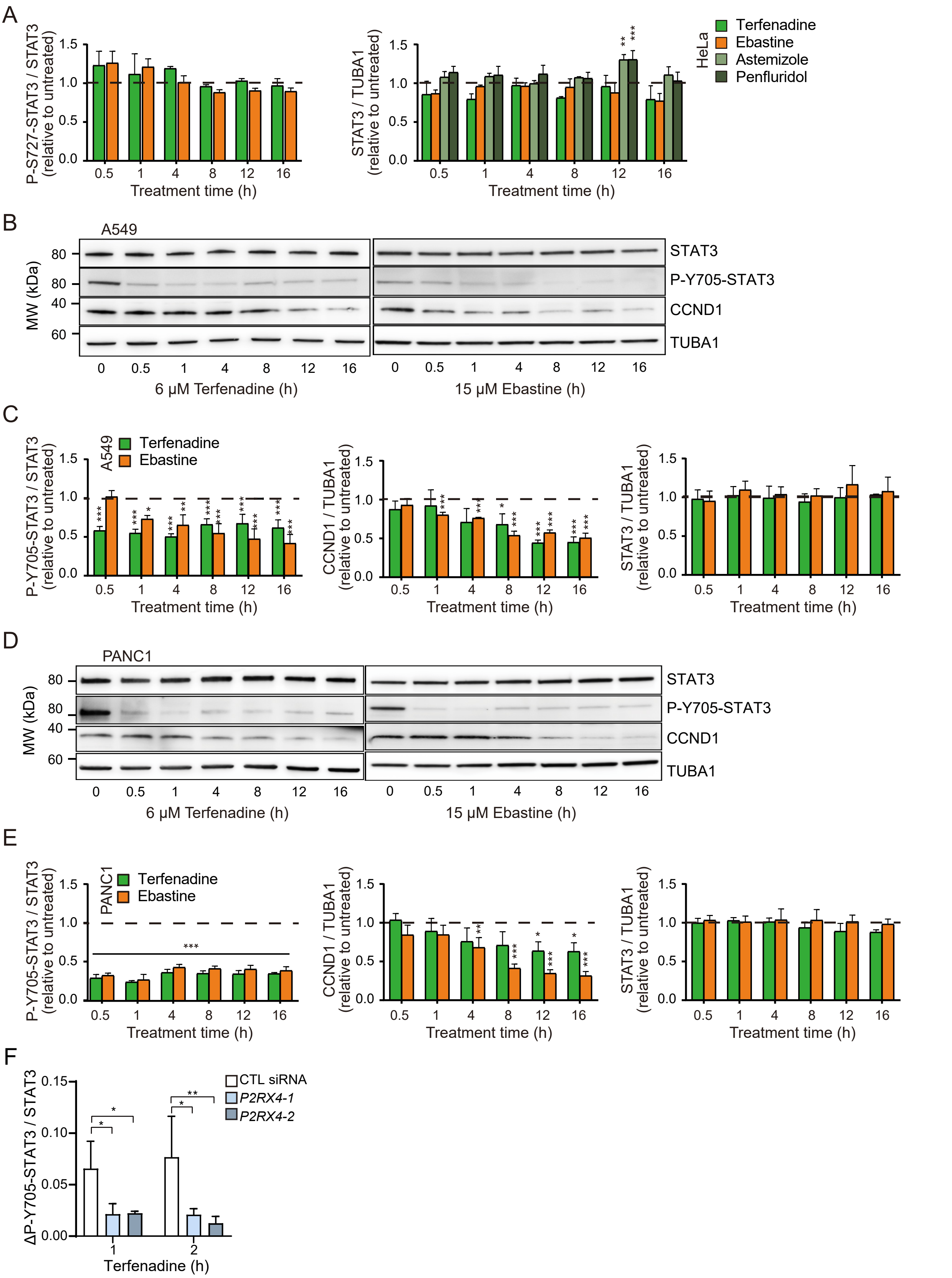
A
P-S727-STAT3 / STAT3 (relative to untreated)
STAT3 / TUBA1 (relative to untreated)
Treatment time (h)
HeLa
Terfenadine
Ebastine
Astemizole
Penfluridol
B
A549
MW (kDa)
STAT3
P-Y705-STAT3
CCND1
TUBA1
6 μM Terfenadine (h)
15 μM Ebastine (h)
C
P-Y705-STAT3 / STAT3 (relative to untreated)
CCND1 / TUBA1 (relative to untreated)
STAT3 / TUBA1 (relative to untreated)
D
PANC1
E
F
ΔP-Y705-STAT3 / STAT3
CTL siRNA
P2RX4-1
P2RX4-2
Terfenadine (h)

Liu et al. Figure S5

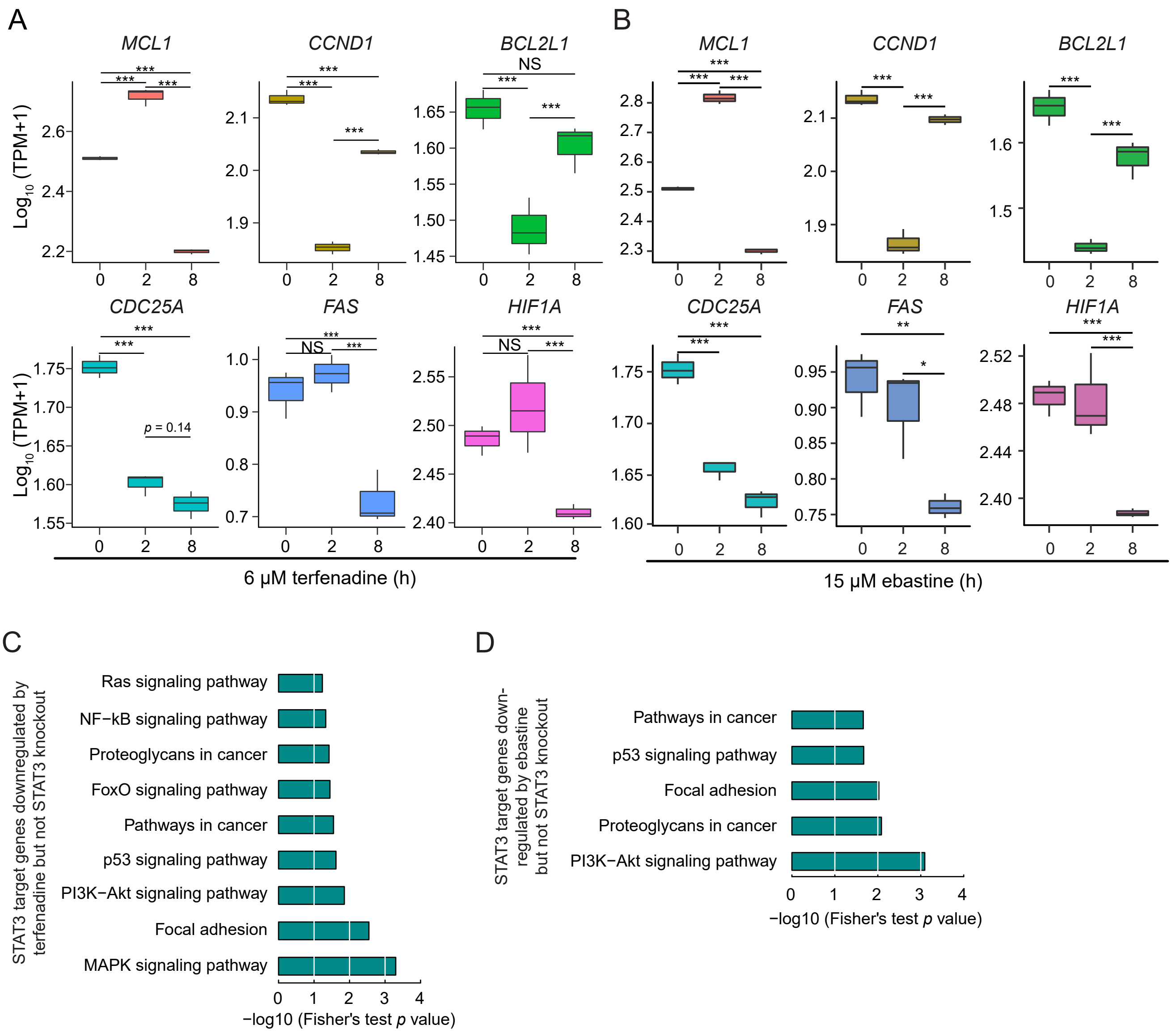

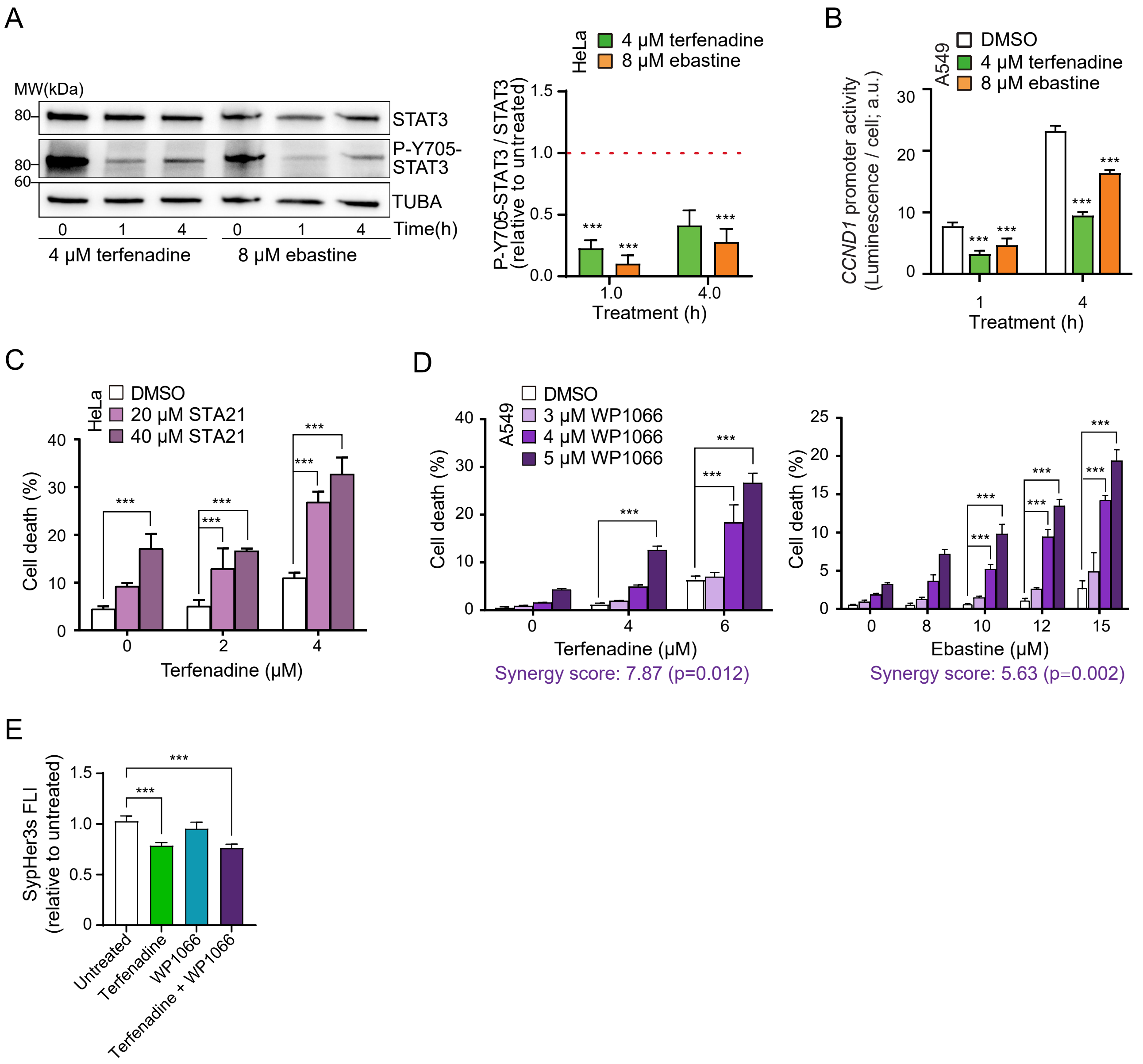
A
MW(kDa)
80
80
60
STAT3
P-Y705-STAT3
TUBA
0 1 4 0 1 4 Time(h)
4 μM terfenadine
8 μM ebastine
HeLa
4 μM terfenadine
8 μM ebastine
P-Y705-STAT3 / STAT3 (relative to untreated)
Treatment (h)
B
A549
DMSO
4 μM terfenadine
8 μM ebastine
CCND1 promoter activity (Luminescence / cell; a.u.)
Treatment (h)
C
HeLa
DMSO
20 μM STA21
40 μM STA21
Cell death (%)
Terfenadine (μM)
D
A549
DMSO
3 μM WP1066
4 μM WP1066
5 μM WP1066
Cell death (%)
Terfenadine (μM)
Synergy score: 7.87 (p=0.012)
Ebastine (μM)
Synergy score: 5.63 (p=0.002)
E
SypHer3s FLI (relative to untreated)
Untreated
Terfenadine
WP1066
Terfenadine + WP1066

Table S1 Oligos and siRNAs

qPCR primers	CCND1S	GAAGATCGTCGCCACCTG
	CCND1AS	GACCTCCTCCTCGCACTTCT
	ACTINS	TGACTTAGTTGCGTTACACCCTT
	ACTINAS	CACCTTCACCGTTCCAGTTTT
Cloning primers	mCherryCASBamHI	gc GGATCC tca CTTGTACAGCTCGTCCATGC
	mCherryNSXhoI	gc CTCGAG ATGGTGAGCAAGGGCGAGGAG
	TMEM165NSKozakNheI	gc GCTAGC gccacc ATGGCGGCCGCGGCTCCAGG
	TMEM165CASXhoI	gc CTCGAG AAAACCAGAATCAGGGCTTAT
	mKeimaNSBamHI	gc GGATCC atggtgagcgtgatcgccaag
	mKeimaCASXbaI	GC TCTAGA TTAGCCCAGCAGGGAGTGGCGG
siRNA	STAT3-1	GAAUCACGCCUUCUACAGA/UCUGUAGAAGGCGUGAUUC
	P2RX4-2	CAAGUCGUGCAUUUAUGAUtt/AUCAUAAAUGCACGACUUGtt
	P2RX4-3	GUCCUCUACUGCAUGAAGAtt/UCUUCAUGCAGUAGAGGACtt
	TMEM165-1	GUAUCUGAAUUGGGUGAUAtt/UAUCACCCAAUUCAGAUACaa
	TMEM165-5	CAGGGUCUAUACAUACUAUtt/AUAGUAUGUAUAGACCCUGaa
	TMEM165-8	GCAUAACAGUACCUCAGAAtt/UUCUGAGGUACUGUUAUGCtt
